# Transcriptomic profiling of the digestive tract of the rat flea, *Xenopsylla cheopis*, following blood feeding and infection with *Yersinia pestis*

**DOI:** 10.1371/journal.pntd.0008688

**Published:** 2020-09-18

**Authors:** David M. Bland, Craig A. Martens, Kimmo Virtaneva, Kishore Kanakabandi, Dan Long, Rebecca Rosenke, Greg A. Saturday, Forrest H. Hoyt, Daniel P. Bruno, José M. Ribeiro, B. Joseph Hinnebusch

**Affiliations:** 1 Laboratory of Bacteriology, Rocky Mountain Laboratories, National Institute of Allergy and Infectious Diseases, NIH, Hamilton, Montana, United States of America; 2 Research Technologies Branch, Rocky Mountain Laboratories, National Institute of Allergy and Infectious Diseases, NIH, Hamilton, Montana, United States of America; 3 Rocky Mountain Veterinary Branch, Rocky Mountain Laboratories, National Institute of Allergy and Infectious Diseases, NIH, Hamilton, Montana, United States of America; 4 Laboratory of Malaria and Vector Research, National Institute of Allergy and Infectious Diseases, NIH, Rockville, Maryland, United States of America; Inserm, FRANCE

## Abstract

*Yersinia pestis*, the causative agent of plague, is a highly lethal pathogen transmitted by the bite of infected fleas. Once ingested by a flea, *Y*. *pestis* establish a replicative niche in the gut and produce a biofilm that promotes foregut colonization and transmission. The rat flea *Xenopsylla cheopis* is an important vector to several zoonotic bacterial pathogens including *Y*. *pestis*. Some fleas naturally clear themselves of infection; however, the physiological and immunological mechanisms by which this occurs are largely uncharacterized. To address this, RNA was extracted, sequenced, and distinct transcript profiles were assembled *de novo* from *X*. *cheopis* digestive tracts isolated from fleas that were either: 1) not fed for 5 days; 2) fed sterile blood; or 3) fed blood containing ~5x10^8^ CFU/ml *Y*. *pestis* KIM6+. Analysis and comparison of the transcript profiles resulted in identification of 23 annotated (and 11 unknown or uncharacterized) digestive tract transcripts that comprise the early transcriptional response of the rat flea gut to infection with *Y*. *pestis*. The data indicate that production of antimicrobial peptides regulated by the immune-deficiency pathway (IMD) is the primary flea immune response to infection with *Y*. *pestis*. The remaining infection-responsive transcripts, not obviously associated with the immune response, were involved in at least one of 3 physiological themes: 1) alterations to chemosensation and gut peristalsis; 2) modification of digestion and metabolism; and 3) production of chitin-binding proteins (peritrophins). Despite producing several peritrophin transcripts shortly after feeding, including a subset that were infection-responsive, no thick peritrophic membrane was detectable by histochemistry or electron microscopy of rat flea guts for the first 24 hours following blood-feeding. Here we discuss the physiological implications of rat flea infection-responsive transcripts, the function of *X*. *cheopis* peritrophins, and the mechanisms by which *Y*. *pestis* may be cleared from the flea gut.

## Author summary

The goal of this study was to characterize the transcriptional response of the digestive tract of the rat flea, *Xenopsylla cheopis*, to infection with *Yersinia pestis*, the causative agent of plague. This flea is generally considered the most prevalent and efficient vector of *Y*. *pestis*. Because most pathogens transmitted by fleas, including *Y*. *pestis*, reside in the insect digestive tract prior to transmission, the transcriptional program induced in the gut epithelium likely influences bacterial colonization of the flea. To determine the specific response to infection, RNA profiles were generated from fleas that were either unfed, fed sterile blood, or fed blood containing *Y*. *pestis*. Comparative analyses of the transcriptomes resulted in identification of 34 infection-responsive transcripts. The functions of these differentially regulated genes indicate that infection of fleas with *Y*. *pestis* induces a limited immune response and potentially alters the insect’s behavior, metabolism, and other aspects of its physiology. Based on these data, we describe potential mechanisms fleas use to eliminate bacteria and the corresponding strategies *Y*. *pestis* uses to resist elimination. These findings may be helpful for developing targeted strategies to make fleas resistant to microbial infection and thereby reduce the incidence of diseases they spread.

## Introduction

Fleas are blood-feeding arthropods that are vectors of several Gram-negative bacterial pathogens including *Bartonella* spp., *Rickettsia felis*, *Rickettsia typhi*, and *Yersinia pestis* [1]. Once ingested by a flea, the bacteria must overcome insect immunity, establish a replicative niche in the gut, and alter their gene expression to promote survival and transmission [2–8]. For *Y*. *pestis*, infection is initiated when fleas ingest blood from a highly bacteremic animal, typically a rodent. Laboratory infection studies estimate that the bacteremic blood must contain ~4x10^7^ CFU/ml of *Y*. *pestis* to result in chronic infection in 50% of the fleas that fed on it [9, 10]. If the blood contains <10^6^ CFU/ml of *Y*. *pestis*, flea infection is rare.

Shortly after ingesting blood, the flea gut initiates peristalsis, mixing the blood meal, free-floating planktonic plague bacilli, and any flea-produced enzymes or antimicrobials that may be present. The bacteria begin to aggregate and a large dense mass is formed in the digestive tract shortly after feeding; the mass appears to adhere to or become ensnared in the numerous spines that line the proventricular valve in the foregut, facilitating initial colonization [3, 11]. Upon sensing the temperature shift from the mammalian to the flea host, the bacilli produce and export an exopolysaccharide that increases the cohesiveness of the aggregate and its adherence to the foregut, resulting in formation of a biofilm [3, 12]. Biofilm masses frequently grow to sizes that would be too large to pass through the lower digestive tract of the flea and be defecated. At any stage of foregut infection, if the bacteria sufficiently obstruct the passage of incoming blood and interfere with the normal function of the proventricular valve, fleas become capable of transmitting *Y*. *pestis* via regurgitation [3, 11, 13].

In Bacot and Martin’s original description of *Y*. *pestis* colonization of the flea, small discrete bacterial aggregates first grow and gradually consolidate into larger masses in the midgut; eventually colonizing the proventriculus over a period of days or weeks [14]. However, we and others have found that when fleas ingest highly bacteremic blood (≥10^8^ CFU/ml *Y*. *pestis*) foregut colonization occurs more rapidly than originally proposed. These masses, containing *Y*. *pestis* and other unknown components, form in the foregut of many fleas within 1 hour of infection and continue to form in others over the next few hours [3, 11, 14]. Biofilm development in many bacterial species enhances resistance to antimicrobials through multiple mechanisms including inhibition of antimicrobial diffusion [15]. In addition, it is likely that flea proteins or byproducts of blood meal digestion (generated and modified by flea enzymes) are incorporated into the biofilm and may play an important role during the initial stages of colonization [3, 11, 12]. Because *Y*. *pestis* likely becomes more refractory to elimination by the flea as the infection progresses, either by digestion, defecation, or the flea immune response, we hypothesize that the flea transcriptional response to infection during the first hours following ingestion and before planktonic plague bacilli aggregate is likely critical for determining whether *Y*. *pestis* can survive, avoid elimination, and perpetuate long-term colonization of the gut. Characterizing these responses may provide insight for developing strategies to prevent infection of the flea vector.

In many arthropods, bacteria that enter the lumen of the gut are exposed to host-produced antimicrobial peptides (AMPs), digestive serine proteases, and reactive oxygen species (ROS) [16]. *Drosophila* and *Galleria mellonella* have recently been adapted as models for *Y*. *pestis*-insect interactions and have been used to show that the bacterial PhoPQ two-component gene regulatory system and the OxyR transcription factor mediate resistance to AMPs and ROS and are required for colonization of *Drosophila* and *Galleria* larvae, as well as the rat flea gut [8, 17, 18]. In studies of flea physiology and genetics, primarily with the cat flea, *Ctenocephalides felis*, many canonical insect responses to pathogens have been observed [7, 19–22]. Recent studies using cultured *Drosophila* cells and RNA interference in cat fleas indicate that *R*. *typhi* infection is moderated by antimicrobials generated by the immune deficiency (IMD) pathway, the major insect innate immune cascade that detects and responds to DAP-type peptidoglycan, common in Gram-negative bacteria [19]. Furthermore, transcriptomic data indicate that the IMD pathway is conserved between *C*. *felis* and the Diptera [19].

Flea immunity and physiology have been characterized mainly using the cat flea model. However, cat flea and rat flea biology differ in important respects. Cat fleas feed frequently and rapidly passage partially digested blood through their gut, likely indicative of distinct physiological and genetic traits that may not be shared with the less-frequently feeding rodent fleas that serve as the primary vectors of *Y*. *pestis* [3, 23]. To address the lack of knowledge about rat flea gut physiology and immunity, we characterized the digestive tract transcription profile of unfed *Xenopsylla cheopis* fleas and of fleas 4h after they had ingested sterile or infected blood. Comparisons among them revealed the flea response following blood feeding in general and to oral infection with *Y*. *pestis* in particular. This analysis resulted in the identification of 23 annotated infection-responsive transcripts that characterize the initial physiological and immunological response of the rat flea gut to challenge with *Y*. *pestis*. The data indicate that upregulation of AMPs by the IMD pathway typifies the initial flea immune response to infection. Expression of genes related to the generation of antibacterial reactive oxygen species (ROS) was limited, suggesting that *Y*. *pestis* does not induce a strong early ROS response by fleas. Genes related to chemosensation, metabolism, and gut peristalsis were differentially expressed, suggesting that feeding behavior and digestive processing are altered following an infected blood meal. In addition, we identified 6 peritrophins produced by *X*. *cheopis* in response to blood-feeding, 3 of which were more highly expressed in infected fleas. Despite the induction of peritrophins, which are frequently integral to formation of a peritrophic matrix (PM), we did not detect convincing evidence for the production of a thick PM during the first 24 hours following feeding, suggesting an alternate role for the *X*. *cheopis* peritrophins.

## Methods

### Flea infection and feeding

Frozen stocks of *Y*. *pestis* KIM6+ were used to inoculate brain-heart infusion (BHI) broth supplemented with 10 μg/ml hemin. Cultures were incubated for 24h, without aeration, at 28°C; 1 ml of this culture was transferred to 100 ml of BHI broth that was then incubated for 18-19h at 37°C. A portion of the bacterial culture, based on OD600 measurements, was centrifuged at 6000 rpm for 10 min and the resulting pellet was resuspended in 1 ml of sterile PBS and added to 5 ml of defibrinated *Rattus norvegicus* blood (BioIVT) resulting in a final concentration of ~5x10^8^ CFU/ml *Y*. *pestis* (S1 Table). Serial dilutions of the infectious blood meal were plated on sheep blood agar to confirm the dose. 5 days prior to oral infection, adult female *Xenopsylla cheopis* fleas were randomly pulled from colonies maintained at Rocky Mountain Laboratories and fed sterile blood through a mouse skin membrane affixed to an artificial feeding device [24]. Any fleas that did not take this pre-infection sterile blood meal were removed from the pool of fleas used for collection of RNA. 5 days later, fleas from this pool were separated into groups and either not fed or allowed to feed on sterile or infectious rat blood. Following feeding, fleas were immobilized and examined using a dissection microscope to confirm that they fed. A sample of 18 to 20 infected fleas was immediately frozen at -80°C for determination of the infectious dose. Fleas were mechanically disrupted in sterile PBS and triturates were plated on BHI soft agar overlays supplemented with hemin and 1μg/ml irgasan. The median CFU/flea for all 6 experiments was 5.2 x 10^4^ (S1 Table).

### Flea dissection and RNA extraction

Four hours after ingesting a sterile or infectious blood meal, fleas were immobilized on ice, placed in a drop of sterile, RNAse-free PBS (Ambion) on a glass microscope slide, and dissected using a pair of fine forceps. After removing the exoskeleton, the flea digestive tract was isolated and transferred to a different, sterile drop of PBS where the Malpighian tubules and hindgut were excised. Finally, the remaining flea midgut and foregut were isolated and transferred to a third drop of PBS where the midgut was gently expressed to remove most of the recent bloodmeal from the gut. Each time flea tissue was transferred between drops of PBS, forceps were rinsed in 70% ethanol and wiped with a Kimwipe. Finally, flea midguts were placed in RNAse-free 1.5 ml Eppendorf tubes containing 200 μl of Kingfisher lysis buffer (Thermo Scientific). Prior to use, freshly prepared dithiothreitol (DTT) was added to the lysis buffer to a final concentration of 40 mM. The process was repeated for pools of 10 fleas in each of the 3 treatment conditions (unfed for 5 days, sterile blood-fed, and infected) and the order that each group was dissected rotated for each of the 6 independent experiments. Dissections for each treatment group required 15–20 min. Immediately following dissections, samples were stored at -80°C for later RNA extraction.

For tissues collected for the peritrophin RT-qPCR screen, fleas were dissected similarly to above except hindgut, Malpighian tubule, and tracheal tissue was also collected and saved for RNA extraction. Tracheal tissue large enough to manipulate with forceps was removed from the midgut as well as separated from the spiracles prior to transferring it to an Eppendorf tube containing lysis buffer. All tissues were collected and pooled from 10 female fleas fed sterile blood 4h prior in 3 independent experiments.

RNA was extracted according to the manufacturer’s instructions (KingFisher Pure RNA Blood kit, Thermo Scientific). Briefly, sample lysates were thawed at room temperature, proteinase K was added, and samples were mixed by inversion and incubated at room temperature for 10 min. Digestion lysate was combined with isopropanol and KingFisher magnetic beads. Samples were incubated at room temperature for 5 minutes and beads were captured on a magnetic stand. Beads were digested with DNase-1 solution, resuspended in rebinding buffer, and washed prior to elution. RNA was eluted and captured from the beads by mixing (900 rpm) and incubating the beads in nuclease-free water at 60°C for 5 min. Purified RNA was evaluated using an Agilent 2100 Bioanalyzer (Agilent Technologies, Palo Alto, CA; S1 Fig) and quantified using spectrophotometer 260 nm absorbance readings.

### Illumina sequencing of *X*. *cheopis* RNA

Eighteen sequencing libraries representing six biological replicates of the three treatments (unfed, blood-fed, and infected blood-fed) were generated from *X*. *cheopis* digestive tracts each using the “Low Sample” (LS) protocol found in the Truseq Stranded mRNA Library Preparation Guide, Revision E (Illumina, San Diego, CA). The barcoded libraries were fragment-sized using a DNA1000 Bioanalyzer Chip (Agilent Technologies, Santa Clara, CA) and quantitated using KAPA Library Quant Kit (Illumina) Universal qPCR Mix (Kapa Biosystems, Wilmington, MA) on a CFX96 Real-Time System (BioRad, Hercules, CA). All samples were pooled together in equimolar concentration and sequenced 100 base-pairs in each read direction on an Illumina HiSeq 2500.

### Bioinformatics and statistical analysis

Assembly of all combined reads was carried out using the Abyss assembler with k values from 25..30..35…95, running in single stranded mode [25]. Subsets of 25 million reads were made for each of the 3 treatments (blood, infected, and unfed) and submitted to the Trinity assembler used in single stranded F mode. Resulting assembly contigs were merged using a BLAST and CAP3 pipeline [26]. Coding DNA sequences (CDS) were extracted based on BLASTx results to Swissp, Genbank insect-derived, and a subset of the non-redundant protein databases. CDS were selected if they began with a methionine, if fragments shared ≥70% similarity with a matching protein, or if they contained a putative signal sequence.

The CDS were used to map the reads from each of the 18 libraries using RSEM/bowtie 2 [27]. To detect statistical differences in expression between the pairwise comparisons listed in Table 1, EdgeR [28] was run on CDS having a fragments per thousand nucleotides per million reads (FPKM) value greater than 5 in at least one of the 18 libraries. A false discovery rate (FDR) of less than 0.05 was considered significant. CDS were compared using BLASTn to an rRNA database to reduce undesirable rRNA in the data set. An automatic annotation program was run that scans the vocabulary of ~400 words and their order of appearance based on the protein matches, including their e-values and coverage. A comparison was made for the highest e-value score for each contig based on their matches to Refseq-Vertebrate, Insecta, and *Yersinia* databases to filter out non-insect CDS from the libraries. Protein sequence alignments were done with the ClustalX package [29]. Phylogenetic trees were done with the MEGA6 [30] package using the maximum-likelihood method with 1000 bootstraps.

**Table 1:** Total number of differentially expressed transcripts between the 3 flea treatment conditions based on statistical analysis.

Comparison	# of Significant Differentially Expressed CDS*
Sterile Blood-Fed vs. Unfed	3881 (1188)
Infected Blood-Fed vs. Unfed	3999 (1253)
Infected Blood-Fed vs. Sterile Blood-Fed	13 (10)
Sterile & Infected Blood-Fed vs. Unfed	4367 (1216)

*The number of transcripts with ≥2-fold change is shown in parentheses.

### Validation of RNA sequencing and RT-qPCR

RT-qPCR probe and primer sets (S2 Table) were designed using Primer Express 3.0 (Life Technologies, Carlsbad, CA). Gene regions that had the greatest number of shared sequence reads across all RNA samples were selected for primer and probe design. Primers and probes were tested against deleterious secondary structures and checked by BLAST alignment for potential cross-hybridization with rat sequences. Template cDNAs were synthesized from the 18 original RNA samples (S1 Fig) using SuperScript VILO cDNA synthesis kit (Invitrogen, Carlsbad, CA). Resulting cDNAs were purified using the QIAquick 96 PCR Purification Kit (Qiagen, Valencia, CA).

Four reference genes were assessed as potential candidates for RT-qPCR normalization: UDP-glucose-glycoprotein-glycosyltransferase isoform X2 (*uggt2*), enolase (*eno*), alpha-aminoadipic-semialdehyde dehydrogenase (*aldh7a1*), and elongation factor 1D (*ef-1d*). Reference gene candidates were ranked and selected based on gene function and by coefficient of variation of normalized reads counts across all RNA samples. Ultimately, *ef-1d* was selected as it had the best PCR amplification efficiency and was expressed at the lowest level. For analysis of peritrophin expression in different tissue types, *eno* and *uggt2* were also used for expression normalization.

RT-qPCR was performed with Invitrogen Express QPCR Supermix Universal with premixed ROX (Life Technologies, Carlsbad, CA). The qPCR reactions were carried out in a 20 μl volume under the following conditions: 50°C for 2 min, 95°C for 2 min, 55 cycles of 95°C for 15 sec, and 60°C for 1 minute. Data was analyzed using 7900HT version 2.4 sequence detection system software (Life Technologies, Carlsbad, CA).

### Peritrophic matrix screen

The midguts dissected from 8 to 10 fleas either immediately or 2, 4, 8, 12, or 24h after feeding on sterile blood were fixed in 4% formalin for 48h. Flea guts from each group were processed using the VIP-6 Tissue Tek processor (Sakura Finetek USA, Torrance, CA), embedded together in Ultraffin paraffin polymer (Cancer Diagnostics, Durham, NC), and sectioned into 4 μm segments. Roughly half of the gut sections were stained with Hematoxylin and Eosin (H&E) and the rest were stained with wheat germ agglutinin, Alexa Fluor 488 conjugate (WGA-AF; Molecular Probes Inc., Eugene, OR) to detect chitin. Slides were deparaffinized with xylene and tissue was rehydrated with successive 5 min ethanol washes (100%, 95%, 70%) with a final 5 min wash with sterile PBS. Slides were stained in the dark at room temperature with 10 μg/ml WGA-AF for 30 minutes. Excess WGA-AF was removed by submerging the slides twice in PBS for 10 minutes with gentle shaking. Slides were tapped dry and gut samples were mounted with Prolong-Gold anti-fade reagent with DAPI and incubated overnight at 4°C prior to imaging. Fluorescent images (12-bit) were acquired using a Zeiss LSM710 laser scanning confocal microscope with a 63x oil/1.4 NA DIC M27 Plan-Apochromat objective using 405 (DAPI) and Argon 488 (AF 488) lasers with emission collection ranges of 410–498 and 493–630, respectively. Light microscopy of H&E stained flea guts was obtained using a Nikon Eclipse E800 microscope and an Olympus DP72 camera with cellSens imaging software.

For transmission electron microscopy, flea guts were dissected 0, 4, 8, 12, or 24h after feeding on sterile blood and fixed overnight at 4°C in 2% paraformaldehyde, 2.5% glutaraldehyde in Sorenson’s phosphate buffer. *Ixodes scapularis* nymph guts were dissected 72h after feeding and fixed and processed identically to flea guts. Flea and tick gut samples were post-fixed for 1h with 1% osmium tetroxide/0.8% potassium ferricyanide in 0.1M sodium cacodylate followed by staining for 1h with 1% tannic acid and post fixation for 1h with 2% osmium tetroxide in 0.1M sodium cacodylate. Next, guts were stained overnight at 4°C with 1% uranyl acetate and then dehydrated in a graded ethanol series and transferred into propylene oxide. Subsequently, the samples were infiltrated with a mixture of 3:1 and 1:1 propylene oxide and EPON/Araldite resin for 1h each under vacuum. The samples were infiltrated with resin overnight under vacuum before being cured in a 68°C oven. Gut samples were sectioned at 80 nm using a UC6 ultramicrotome (Leica Microsystems, Vienna, Austria). Images were collected using a Hitachi HT7800 transmission electron microscope (Hitachi, Tokyo, Japan) at 80kV.

### *In situ* hybridization

Fleas were fed infectious rat blood containing *Y*. *pestis* as described above. 4 hours after feeding, groups of 24 fleas were collected and immobilized, the flea cuticle was punctured 3–4 times using an insect pin, and whole fleas were immediately placed in a modified Carnoy’s fixative (60% ethanol, 30% chloroform, 10% formaldehyde) and incubated at 4°C overnight. Groups of fixed fleas were processed using the VIP-6 Tissue Tek processor (Sakura Finetek USA), and then embedded in paraffin. Blocks were sectioned at 5 μm and slides were counterstained with hematoxylin. ISH was performed using the RNAscope VS universal assay and the Basecope VS reagent kit (Advanced Cell Diagnostics, Newark, CA) on the Roche/Ventana Discovery ULTRA staining platform (Roche Tissue Diagnostics, Indianapolis, IN). Briefly, unique RNAScope probes bind to target RNA sequences (in fixed flea tissues) in tandem, creating a unique binding site for the pre-amplifier reagent to bind. The amplifier reagent then binds to pre-amplifier reagent and target RNA is detected chromogenically by interaction of alkaline phosphatase with the Fast Red substrate [31]. The complete sequence for each of the transcripts was provided to Advanced Cell Diagnostics for synthesis of custom RNAscope (trypsin-like serine protease and Ajuba) or BASEscope (peritrophin A and B) probes. Flea sections were screened for tissue specificity and staining intensity.

## Results and discussion

### Transcriptional profile of the flea gut in response to blood feeding and to infection with *Y*. *pestis*

**Fig 1:**
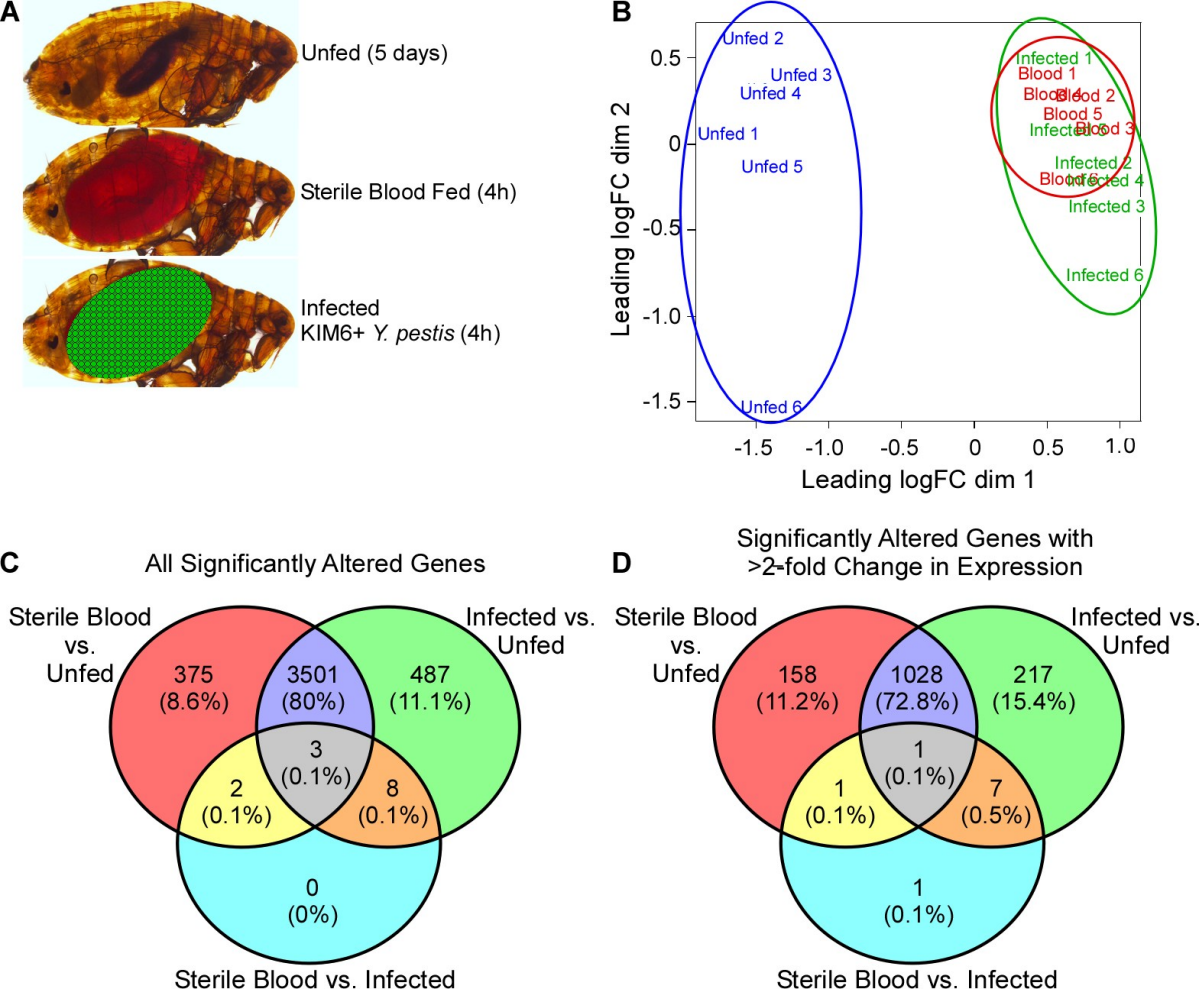
Digestive tract transcriptional profiles of unfed, sterile blood-fed, and infected blood-fed *X*. *cheopis* fleas. A) Outline of the three flea treatment groups used for RNA-seq analysis: 1) unfed fleas; 2) fleas fed sterile rat blood; 3) fleas fed infected rat blood (fleas ingested an average of 7x10^4^ *Y*. *pestis* CFU). B) Multidimensional scaling (MDS) plot of the 6 replicate gene expression profiles for each of the 3 groups: unfed (blue), blood-fed (red), infected (green). Venn diagrams representing C) the number of transcripts with significantly altered expression for 3 comparisons between flea treatment groups (see Table 1) and D) only those transcripts with ≥2-fold change in expression.

To assess the transcriptomic response of the flea gut to oral infection, digestive tracts (proventriculus + midgut) were isolated from female *X*. *cheopis* in 3 different treatment groups: 1) not fed for 5 days; 2) fed sterile rat blood; or 3) fed rat blood containing ~5x10^8^ CFU/ml *Y*. *pestis* KIM6+ (Fig 1A). Guts were dissected and pooled from 10 fleas from each group 4h after feeding (except for unfed fleas) and this process was repeated in 6 independent experiments. mRNA was extracted from pooled digestive tract samples (S1 Fig), next-generation RNA sequencing was performed, and transcript libraries were assembled *de novo*. Rat and *Yersinia* transcripts, as well as contaminating rRNA sequences, were removed from the assembly. The multidimensional scaling plot (MDS) of transcript profiles showed distinct clustering for the unfed flea samples, while blood-fed and infected flea transcriptomes clustered similarly (Fig 1B). The MDS plot indicates that ingestion of blood induces most of the changes in transcript expression between fed and unfed fleas.

**Fig 2:**
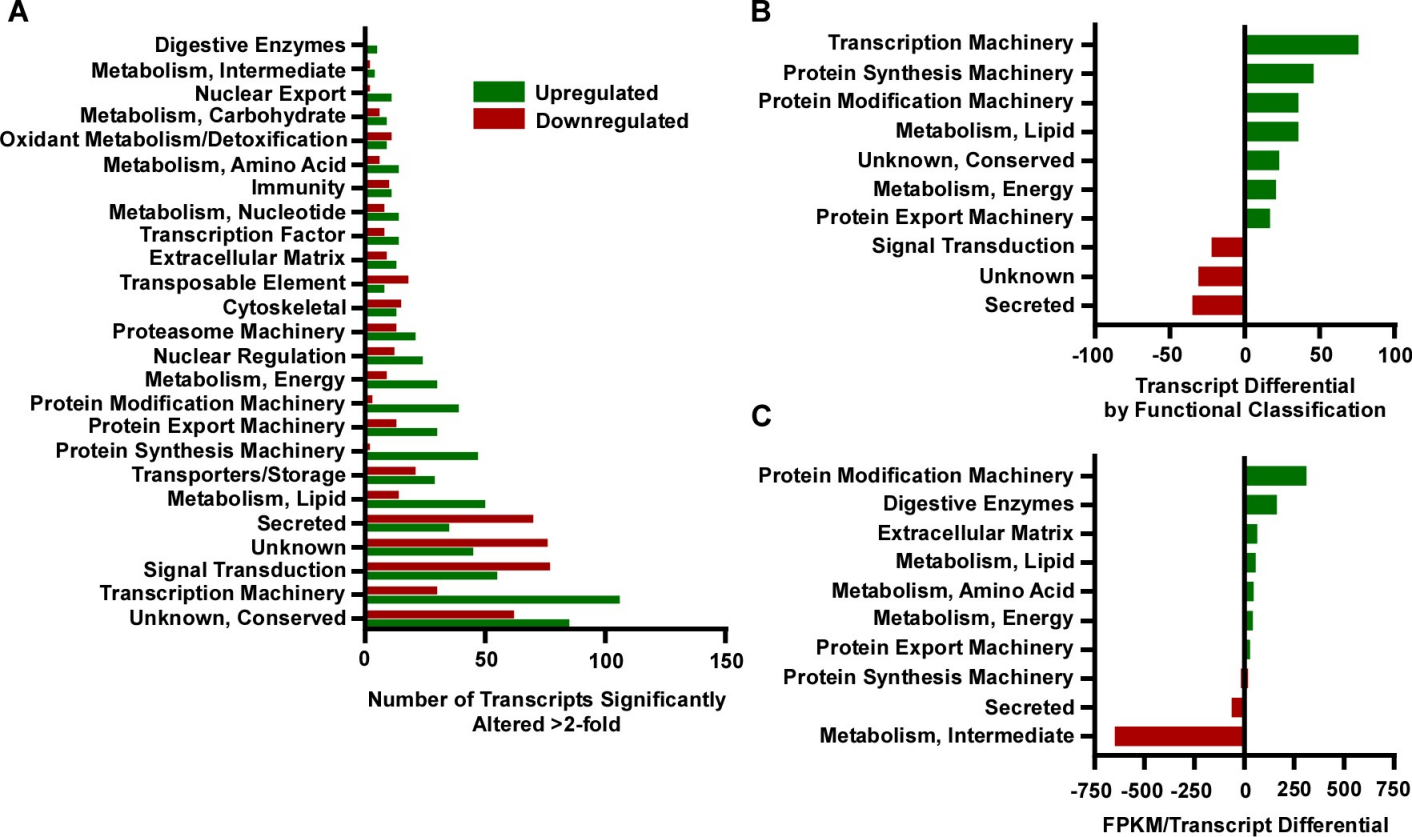
Summary of *X*. *cheopis* digestive tract genes up- or down-regulated in response to sterile blood-feeding and infection. A) Functional categorization of genes with significantly (≥2-fold) up- or down-regulated expression in response to both sterile blood-feeding and infection (n = 1216). B) The difference in the number of transcripts that were up- and down-regulated within various functional classifications. C) The difference in transcript abundance ((average FPKM for all upregulated transcripts)–(average FPKM for all downregulated transcripts)) within various functional classifications. For B and C, functional classifications with the 10 largest differences are shown.

Bioinformatic analysis identified 8080 coding DNA sequences (CDS) in the flea digestive tract samples. 3881 to 4367 of these were significantly up- or down-regulated in infected and sterile blood-fed fleas relative to the unfed group (Fig 1C and Table 1), and 1188 to 1253 of these were altered by at least 2-fold (Fig 1D and Table 1). Each of the 1216 transcripts differentially expressed by at least 2-fold in response to blood feeding and infection was assigned a functional classification based on annotation, and the 721 upregulated and the 495 downregulated transcripts were organized in a histogram (Fig 2A). Genes involved in transcription machinery, protein synthesis, and protein modification were the most overrepresented upregulated functional categories while genes involved in signal transduction, protein secretion, and with unknown functions were the most overrepresented downregulated categories (Fig 2B). However, when contigs were weighted for the relative abundance of RNA per transcript (as determined by fragments per kilobase of transcript per million mapped reads; FPKM), the most overrepresented upregulated functional categories were those involved in protein modification machinery, digestion, extracellular matrix production, and lipid metabolism; in contrast, secreted transcripts and those involved in intermediate metabolism were primarily downregulated (Fig 2C).

Most of the upregulated transcripts involved in protein modification encoded heat shock proteins or molecular chaperones, including 7 of the 25 most abundant transcripts (S3 Table). The rapid ingestion of large amounts (relative to their body size) of warm blood by arthropods induces mechanical, thermal, osmotic, and oxidative stress in the gut [32]. To deal with these stresses, arthropods produce heat shock proteins to moderate their influence and regulate the reparation of any damage or dysfunction they induce [32]. These proteins are also involved in the proper folding of newly synthesized proteins. Most of the upregulated digestive enzyme genes encode trypsins or trypsin-like proteins, serine proteases that convert proteins into smaller peptides during digestion. However, the most prominent metabolic category altered in response to blood ingestion were genes involved in the digestion and metabolism of lipids (Fig 2B and 2C). An acid sphingomyelinase, which catalyzes the breakdown of sphingomyelin phospholipid into ceramide and phosphorylcholine [33], was the most abundant upregulated lipid metabolism transcript (S3 Table). Conversely, fatty acid synthase was the most abundant downregulated lipid metabolism transcript. The expression pattern of fatty acid synthase, along with the upregulation of enoyl-CoA hydratase and lipid storage droplet transcripts, suggest that fleas utilize blood meal-derived fatty acids for both energy production and storage (S3 Table). The majority of the upregulated extracellular matrix production genes encoded peritrophins, one of which was the 21^st^-most abundant upregulated transcript (S3 Table). The expression of peritrophin transcripts was unexpected as fleas are not thought to produce a peritrophic matrix (PM), of which peritrophins are an important structural component [34, 35]. The PM is a semi-permeable acellular sheath, composed of proteins, glycoproteins, proteoglycans, and interwoven chitin fibrils, that surrounds the arthropod blood meal following feeding and prevents direct contact of blood with the gut epithelium [34].

### Transcriptional changes in response to infection

**Fig 3:**
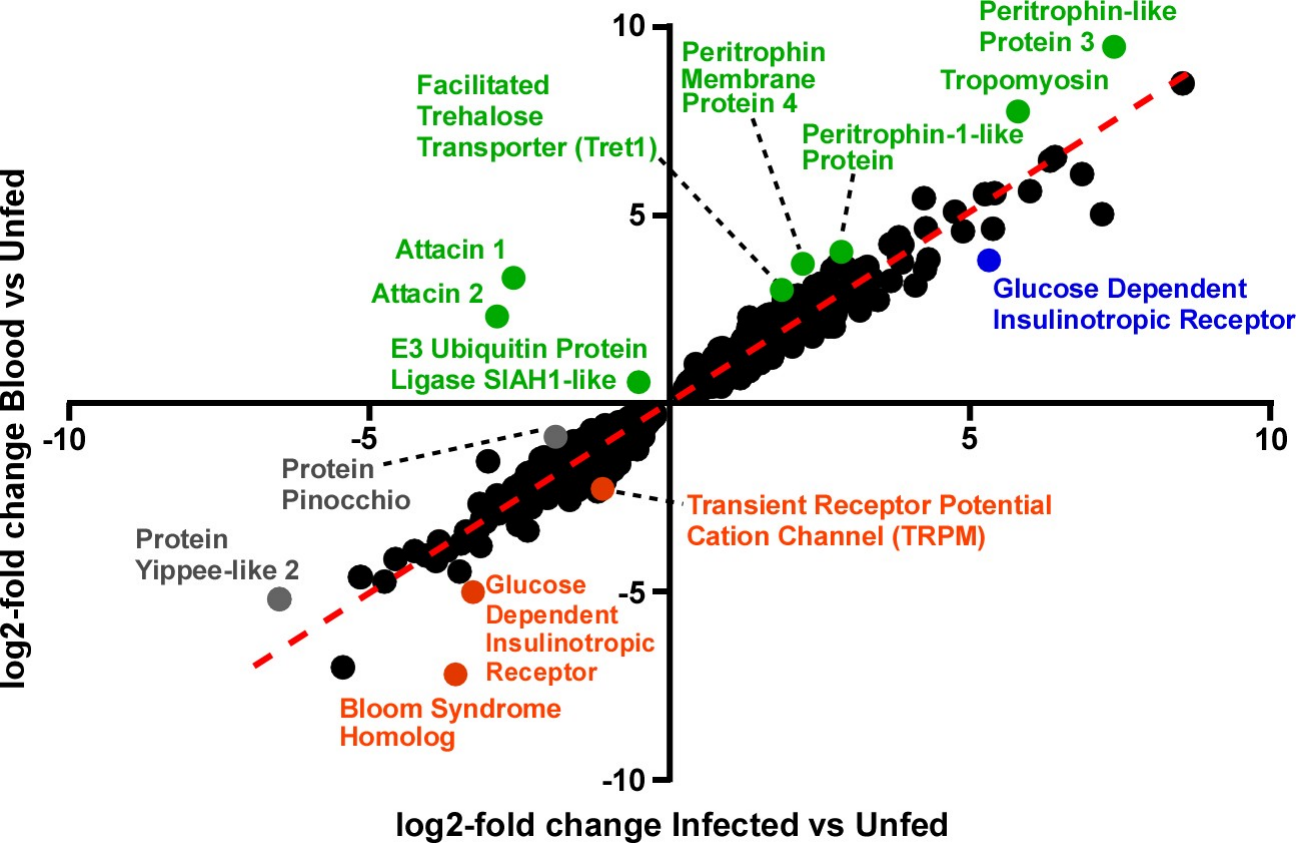
Infection-responsive transcripts identified by expression differentials in sterile blood-fed and infected fleas compared to unfed fleas. Scatter plot of 2-fold change values for all transcripts (n = 3501) that were significantly altered in both the sterile blood-fed vs unfed (x-axis) and unfed vs. infected blood-fed (y-axis) statistical comparisons (Fig 1A and Table 1). The 14 annotated outlier transcripts (≥2-fold change differential) that were more upregulated in infected fleas (green symbols), more downregulated in infected fleas (orange symbols), more upregulated in sterile blood fed fleas (blue symbol), or more downregulated in sterile blood fed fleas (grey symbols) are labeled. These transcripts are listed in Table 3.

We used two approaches to determine infection-responsive flea transcripts: 1) statistical comparison of altered transcript expression between fleas fed sterile blood and those fed an infectious blood meal (Table 2); and 2) identification of outlier transcripts by calculating expression differentials for two comparisons: sterile blood-fed fleas vs. unfed controls and infected fleas vs. unfed controls (Fig 3 and Table 3).

**Table 2:** Infection-responsive transcripts identified by statistical comparison.

Contig Name	Encoded Protein	Fold Change	E Value	Coverage (%)	Protein Database
Xc2751	Attacin	60.8	7e-033	98	INSECTA
Xc19158	Attacin 2	35.8	2e-034	111	INSECTA
XcSigP-1091	Coleoptericin-like	10.9	7e-006	97	INSECTA
XcSigP-10967	14.3 kDa Midgut Protein	3.6	2e-007	62	INSECTA
Xc26939	Constitutive Coactivator of Peroxisome Proliferator-Activated Receptor Gamma-like (CCPG)	3.3	1e-55	95	INSECTA
Xc69039	Relish	2.2	1e-123	113	INSECTA
Xc72760	E3 Ubiquitin-Protein Ligase SIAH1-like	2.1	6e-25	99	INSECTA
Xc75526	Sulfide:Quinone Oxidoreductase	1.9	1e-166	95.7	KOG
XcSigP-75988	Chymotrypsin-2-like	1.9	1e-11	27	INSECTA
Xc44801	O-acetyl-ADP-Ribose Deacetylase 1	1.5	6e-33	81	SWISSP
XcSigP-30091	Putative Allatostatin C	-2.7	2e-17	96	INSECTA
XcSigP-22740	General Odorant-Binding Protein 56D	-8.4	1e-17	96	INSECTA

**Table 3:** Infection-responsive transcripts identified by outlier expression differential.

Contig Name	Encoded Protein	Log2-Fold Change Differential (Δ≥1)*	E Value	Coverage (%)	Protein Database
**More up- or down-regulated in infected fleas**					
XcSigP-32200	Peritrophin-like Protein 3	2.1	3e-010	98	INSECTA
Xc71916	Tropomyosin isoform X2	1.9	0	102	REFSEQ-INVERTEBRATE
Xc26330	Peritrophic membrane protein 4	1.5	6e-025	46	INSECTA
XcSigP-1797	Peritrophin-1-like Protein	1.2	8e-010	69	INSECTA
XcSigP-23061	Facilitated Trehalose Transporter (Tret1)	1.1	0	74	REFSEQ-INVERTEBRATE
XcSigP-73090	Transient Receptor Potential Cation Channel (TRPM)	-1.2	3e-092	13	INSECTA
XcSigP-75957	Glucose-Dependent Insulinotropic Receptor	-1.8	6e-26	70	INSECTA
Xc72063	Bloom Syndrome Homolog	-3.7	0.013	23	REFSEQ-INVERTEBRATE
**More up- or down-regulated in sterile blood-fed fleas**					
XcSigP-74348	Glucose-Dependent Insulinotropic Receptor	1.5	3e-26	60	INSECTA
Xc23722	Protein Pinocchio	-1.0	4e-035	75	INSECTA
XcSigP-12487	Protein Yippee-Like 2	-1.3	9e-062	82	INSECTA

* (log2 fold change Sterile Blood vs. Unfed)–(log2 fold change Infected Blood vs. Unfed)

The first approach revealed 12 annotated transcripts that were significantly altered in the sterile blood-fed vs. infected flea comparison (Table 2). Because the vast majority of transcripts were expressed in a similar pattern by infected and sterile-blood fed groups (Figs 1B and 3), we used the second approach to identify additional infection-responsive transcripts by plotting the log2-fold change expression values for transcripts that were significantly altered in both sterile blood-fed vs. unfed and infected blood-fed vs. unfed flea comparisons. Transcripts that had a differential of ≥ 1 log2-fold change between the two comparisons were considered outliers (Fig 3). This resulted in identification of an additional 11 unique transcripts (~0.3% of the 3501 transcripts analyzed) with at least 2-fold difference in expression between the two conditions relative to the unfed, baseline fleas (Table 3). Three of the outlier transcripts more highly expressed in infected fleas (attacin 1 & 2; E3 ubiquitin-protein ligase) were identified using both approaches (Tables 2 and 3, Fig 3). Of the newly identified contigs, 5 were more upregulated in infected fleas (green symbols; Fig 3 and Table 3), 3 were more downregulated in infected fleas (orange symbols; Fig 3 and Table 3), 1 was more upregulated in sterile blood-fed fleas (blue symbol; Fig 3 and Table 3), and 2 were more downregulated in sterile blood-fed fleas (grey symbols; Fig 3 and Table 3). Eleven infection-responsive transcripts that encoded unknown or uncharacterized genes were also identified and are listed in the supplemental material (S4 Table) but not discussed or plotted on the outlier graph (Fig 3).

To confirm results from the data set, a panel of blood and infection-responsive flea transcripts was selected for validation by RT-qPCR. Expression trends of the 6 transcripts closely matched the results from our RNA-seq data set (S2A Fig). Linear regression analysis of RNA-seq and RT-qPCR expression levels showed good correlation between the two methods (R^2^ = 0.849; S2B Fig).

In summary, the two approaches resulted in the cumulative identification of 23 annotated infection-responsive genes (Tables 2 and 3) corresponding to four functional themes that characterize the early transcriptional response of fleas to ingestion of *Y*. *pestis*: 1) production of antimicrobial peptides and upregulation of the immune response; 2) regulation of chemosensation and gut peristalsis; 3) modification of digestion and metabolism; and 4) production of chitin-binding proteins (peritrophins). These will be discussed in turn in the following sections.

### The flea immune response to oral infection with *Y*. *pestis*

The most highly upregulated infection-responsive transcripts in our analysis encode 3 antimicrobial peptides (AMPs): two attacins and a coleoptericin-like peptide whose expression increased 11- to 61-fold in response to *Y*. *pestis* (Table 2). Although grouped together with antimicrobial peptides, attacins are relatively large proteins (~20 kDa) that bind to the LPS of Gram-negative bacteria, increase membrane permeability, and inhibit synthesis of outer membrane proteins [36, 37]. Coleoptericins act on both Gram-negative and -positive bacteria and are hypothesized to interact with the GroEL chaperone to elicit bacteriostatic and bactericidal function [38–40]. *Y*. *pestis* in the flea gut is generally thought to be resistant to AMPs, partially due to temperature-dependent modification to its lipid A structure and other cell envelope adaptations moderated by the PhoPQ two-component gene regulatory system [41–43]. Studies of flea antibacterial responses and minimum inhibitory concentrations of *Y*. *pestis* to cationic antimicrobials suggest fleas may be unable to meaningfully control oral infection with plague bacilli [7, 42, 44]. However, insect antimicrobial peptides can work synergistically to control bacterial infection in the gut [45], as evidenced by observations of relatively high AMP minimum inhibitory concentrations (MIC) for many bacterial species when tested individually, but lower MICs when tested combinatorially in *in vitro* susceptibility assays [46]. Furthermore, most *Y*. *pestis*-flea infection studies, including this one, utilize relatively high infectious doses in the bloodmeal (≥10^8^ CFU/ml). Lower flea challenge doses (≤1x10^7^ CFU/ml), which are probably more ecologically and physiologically relevant, may be easier for the flea immune system to manage [9]. Relevant to this point, our study identified 2 lysozyme transcripts that were upregulated in response to a sterile or infectious blood meal, which may contribute to maximal potency of infection-responsive AMPs (Table 4). *Y*. *pestis* produces a periplasmic lysozyme inhibitor (Ivy) that provides lysozyme resistance for bacteria grown at mammalian temperature (37°C), but not flea temperature (21°C) [47]. While Ivy expression is repressed in the flea, recently ingested bacteria still conditioned to the mammalian host phenotype may utilize Ivy to resist lysozyme during the early stages of flea colonization [48]. *Yersinia* spp. produce another putative lysozyme inhibitor (Plil) that confers tolerance to invertebrate-type lysozyme [49].

**Table 4:** Selected immunity genes differentially expressed in response to feeding and infection.

Contig Name	Encoded Protein	Fold Change	E Value	Coverage (%)	Protein Database
Xc45094	Cactus	10.3	4e-067	101	INSECTA
XcSigP-40662	Lysozyme 2	5.2	7e-033	86	NR-LIGHT
XcSigP-71044	Peptidoglycan Recognition Protein LB	1.9	7e-054	80	REFSEQ-INVERTEBRATE
Xc73488	Dual Oxidase Maturation Factor	1.4	0	91	INSECTA
Xc71673	Peptidoglycan Recognition Protein LC	1.3	6e-041	90	INSECTA
Xc27529	Lysozyme 1	1.7	2e-48	87	INSECTA
XcSigP-75613	Defensin A	1.8	7e-6	101	SWISSP

Arthropods typically have two major pathways that govern innate immune responses to microbes in the gut: the immune deficiency pathway (IMD) that detects and responds primarily to Gram-negative peptidoglycan; and Toll, which responds to Gram-positive bacteria and fungi [16, 50]. Relish, an NF-κB-like transcription factor that controls most gene expression in the IMD regulon [51], was upregulated in infected fleas, coinciding with upregulation of the AMP transcripts (Table 2). To upregulate the IMD pathway, meso-diaminopimelic acid (DAP)-type peptidoglycan is detected principally through the peptidoglycan recognition protein LC (PGRP-LC) transmembrane receptor [52], which was modestly upregulated in sterile blood-fed and infected fleas (Table 4). PGRP-LB, which negatively regulates the IMD pathway, was up-regulated in response to both sterile and infected blood meals. Ubiquitination is critical for signaling and repression of the IMD pathway, which may partially explain why an E3-ubiqutin ligase was also upregulated in the infected flea gut (Table 2) [53, 54].

Although not necessarily part of the IMD regulon, our screen also identified a Yippee-like protein as being more highly expressed in infected fleas (Table 3). Yippee-like proteins are upregulated in response to bacterial challenge and may interact with moth hemolin, an immunoglobulin-like protein that detects bacterial lipopolysaccharide in the hemocoel of silkworm larvae [55, 56].

The negative suppressor of the Toll pathway, Cactus, was upregulated in response to both infected and sterile blood meals; indicating that, as anticipated, Toll is not involved in the response to *Y*. *pestis* (Table 4). Concurrently, several antimicrobial gene transcripts with potential activity against Gram-positive bacteria, such as lysozyme 1 and defensin A were also downregulated. The expression pattern of *X*. *cheopis* AMPs, PGRPs, and IMD and Toll pathway mediators are indicative of a genetic program that screens ingested blood for pathogen-associated molecular patterns (PAMPs) each time the flea feeds and rapidly produces a targeted antimicrobial repertoire when needed. Furthermore, the data suggest that the Toll pathway plays a limited role, if any, in moderating *Y*. *pestis* infection.

Although several transcripts related to oxidant metabolism and detoxification were identified, few had greater than 2-fold change in expression for any of our comparisons. Most of these transcripts encoded cytochrome P450 oxygenases and were downregulated in response to feeding (S5 Table). Catalase, which converts hydrogen peroxide into water and oxygen, was constitutively expressed in the flea gut and was slightly downregulated in response to a blood meal. Only one transcript related to the microbial-responsive dual-oxidase (DUOX) pathway (dual oxidase maturation factor; Table 4), which produces ROS in response to microbial challenge in *Drosophila*, was differentially expressed and it was only modestly upregulated. In addition, no DUOX NADPH oxidase transcripts were detected in the flea transcriptome [57]. This was unexpected as ROS are common products of the arthropod innate immune response and levels of peroxide in the flea gut increase 3h following infection with *Y*. *pestis* [7, 58]. It is possible that our selected time point (4 hr post feeding/infection) was too early (or late) to capture other transcripts related to ROS production.

Genes related to ROS production are implicated in controlling *Y*. *pestis* levels in the gut of the *X*. *cheopis* [7]. Furthermore, *Y*. *pestis* mutant strains predicted to have regulatory defects that render them more susceptible to ROS are not as efficient at early colonization of the flea gut and can be outcompeted by parental strains in co-infection experiments [7, 8]. Because fleas rapidly digest their blood meal, it is possible that the gene products and processes that break down red blood cells induce the concurrent release of high concentrations of pro-oxidizing heme, which contains iron and can generate ROS. Thus, the release of free heme may generate ROS that inhibit early bacterial colonization of the flea gut [59, 60]. Another factor to consider is that the hemoglobin of certain rodents crystalizes in the flea gut shortly after ingestion [13]. Brown rat blood (which was used to feed the fleas used in this study), is poorly soluble in the flea gut and forms numerous oxyhemoglobin crystals which may not have the same potential to generate ROS as solubilized heme [13]. Some blood-feeding arthropods, such as kissing bugs, promote formation of hemozoin, a chemically inert hematin crystal, in their gut as a mechanism of protection from the toxic oxidative effects of heme [59, 61]. Hemoglobin crystals in the flea gut do eventually solubilize, however the rate of their solubilization, and by association, the source of the flea host blood, may greatly influence the kinetics of ROS production in the gut. This may be another explanation for why more ROS detoxification genes were not observed in our RNA-seq analysis.

### Regulation of chemosensation, behavior, and gut peristalsis

Odorant binding proteins (OBPs) are ligand-binding proteins generally thought to be involved in chemosensory behavior and are expressed in multiple tissues, including those not solely dedicated to olfaction [62]. OBP56d is expressed in both gustatory and olfactory sensilla in *Drosophila* [63], and in our analysis was 8-fold downregulated in infected fleas (Table 2). OBP56a is important for detection of the microbial odorant geosmin and induces avoidance behavior and inhibits positive chemotaxis and feeding [64]. In tsetse flies, OBP6 is needed for development of crystal cells which are important for the melanotic immune response and may function as a carrier protein of microbial associated molecular patterns in order to stimulate immune cell development [65].

In contrast, the gene encoding protein Pinocchio, *smi21F* (a smell-impaired locus) in *Drosophila*, was more highly expressed in infected fleas (Fig 3). Pinocchio was originally identified in fruit fly antennae and was shown to modulate olfactory avoidance and attractant responses [66]. Interestingly, *smi21F* mutant *Drosophila* have an overall upregulation of immune related transcripts including AMPs, proteolytic enzymes, and odorant binding proteins compared to wild-type flies [67]. These observations led to the hypothesis that Pinocchio may contribute to early recognition and removal of small hydrophobic odorants by both the insect olfactory and immune systems.

Members of the transient receptor potential ion channel (TRP) superfamily are typically calcium and/or magnesium permeable transmembrane channels that play a variety of roles in cell signaling [68, 69]. An *X*. *cheopis* TRPM gene was more downregulated in fleas infected with *Y*. *pestis* (Table 3). The sole *Drosophila* gene in the TRPM family is needed for regulation of cell size and zinc homeostasis [70, 71]. Regulation of metal homeostasis is important for midgut physiology, and zinc is an essential micronutrient required for correct folding and activity of various enzymes [72]. However, in *Drosophila*, most TRPs are involved in regulating a variety of sensory mechanisms, including olfaction and gustation, and the resulting changes in behavior [70]. Based on typical TRP function, the expression pattern, and the relatively low coverage of this flea transcript we placed it in the chemosensation functional category.

Allatostatins are pleiotropic neuropeptides that have myoregulatory function in the insect gut [73]. C type allatostatins have been demonstrated to inhibit midgut peristalsis and feeding in moth larvae [74, 75]. The flea gut is muscular and, shortly after feeding, begins peristalsis; churning the ingested blood and likely enhancing the rate of digestion [3, 13]. The proventriculus is also muscular and opens and closes to mechanically disrupt red blood cells [76]. Allatostatin C is downregulated in infected fleas, possibly increasing the frequency of muscle contractions in the gut (Table 2). Increasing peristalsis and the rate of blood meal processing may allow infected fleas to more quickly defecate bacteria present in the gut. This may also explain why a tropomyosin isoform, critical for muscle contraction, was more highly expressed in infected fleas (Table 3).

A transcript annotated as glucose-dependent insulinotropic receptor (GIPR) was identified twice in our screen, in one instance being more highly downregulated in infected fleas relative to unfed fleas and in the other more upregulated in blood-fed fleas (Table 3). Insulin-signaling cascades are generally well conserved among model organisms, including *Drosophila*, where insulin signaling plays important and diverse regulatory roles in behavior, development, aging, immunity, metabolism, and stress responses [77, 78]. In *Anopheles stephensi*, insulin signaling in the midgut is altered in response to an infectious blood meal containing *Plasmodium* or heat-killed *E*. *coli*, resulting in increased host-seeking and feeding behavior for a subset of mosquitoes [79]. In addition, *Plasmodium* induces insulin-like peptide expression in the mosquito gut which, in turn, moderates NF-κB signaling and suppresses expression of IMD immune effectors [80]. Furthermore, blood meal derived insulin can trigger nitric oxide production in the *A*. *stephensi* midgut lumen to control malaria parasite development [81]. The *X*. *cheopis* insulin receptor transcripts could plausibly be involved in up to 3 of our infection-response functional themes including immunity, metabolism, as well as chemosensation and behavior.

Cumulatively, these data suggest that chemosensation is altered in response to infection with *Y*. *pestis* and that infected fleas may display altered behavioral patterns (attraction, avoidance, feeding) and rates of peristalsis within a few hours after an infected blood meal.

### Digestion and metabolism

Serine proteases (trypsin and chymotrypsin) that convert proteins into smaller peptides are the most commonly produced digestive enzymes in blood-feeding arthropods and fleas are no exception [82]. However, some serine proteases are upregulated in cat fleas challenged with *R*. *typhi* and rat fleas challenged with *Y*. *pestis* [7, 22]. In our analysis, a putative chymotrypsin 2-like serine protease was significantly upregulated in response to infection with *Y*. *pestis* (Table 2). This trypsin-like protein lacks an amino-terminal Clip domain. The presence of a Clip domain is typically observed in non-digestive serine proteases involved in protein-protein interactions such as regulation of the prophenol-oxidase cascade or activation of the Toll receptor ligand Spaetzle [83].

Constitutive coactivator of peroxisome proliferator activated receptor gamma (CCPG) is a coactivator that enhances transactivation of the peroxisome proliferator activated receptor gamma transcription factor (PPARγ), implicated in a diverse array of physiological processes, including adipogenesis, fatty acid storage, and glucose metabolism [84, 85]. Increased activation of PPARγ (via upregulation of CCPG) likely indicates alterations to lipid metabolism as a result of oral *Y*. *pestis* infection (Table 2). Interestingly, lipid metabolism was among the most upregulated functional classifications in response to a sterile or infectious blood meal by both the number of differentially regulated transcripts and by transcript abundance (Fig 2A–2C). Aspects of lipid metabolism may play a critical role in flea colonization and transmission, because the biofilm is enveloped in a complex waxy-looking extracellular matrix in order to colonize the proventriculus [3]. In addition, *Y*. *pestis* produces a phospholipase D enzyme, hypothesized to provide protection against a bacteriolytic byproduct of blood meal digestion, which greatly enhances its ability to produce chronic infection of the flea gut [4].

A sugar transporter, annotated as facilitated trehalose transporter 1 (TreT1), was more highly expressed in infected fleas (Table 3). Trehalose is the predominant sugar in the hemolymph of insects, where it serves as a source of energy and provides protection from heat stress and desiccation [86, 87]. In the insect fat body, Tret1 transports trehalose to the hemolymph. Although trehalose and Tret1 are thought to be produced primarily in the insect fat body, transcripts with similarity to Tret1 are frequently expressed by insect midgut epithelium [88, 89]. Interestingly, trehalose, which is hydrolyzed by trehalase to produce two glucose molecules, is the first sugar utilized in the insect chitin biosynthesis pathway [35, 90].

Two transcripts involved in energy production and mitochondrial metabolism were upregulated in infected fleas: sulfide:quinone oxidoreductase and O-acetyl-ADP-ribose deacetylase (Table 2). Upregulation of energy production suggests that ingestion of *Y*. *pestis* imposes an energetic cost to infected fleas, which must produce additional ATP in order to respond to the bacteria.

One transcript that did not fit within the rubric of our flea infection physiological themes was Bloom Syndrome protein. This protein is a member of the RECQ family of DNA helicases and is important for DNA repair and recombination in *Drosophilia* [91, 92]. It was downregulated in infected fleas and its expression pattern suggests that *Y*. *pestis* infection may possibly reduce the ability of the midgut epithelium to repair damage to its DNA (Table 3). Alternatively, *Y*. *pestis* infection may actively be reducing overall levels of oxidative stress, resulting in decreased damage to epithelial cell DNA and thus a downregulation of Bloom Syndrome protein [7].

### Peritrophins (chitin-binding proteins)

Shortly after feeding, the midgut epithelium of many blood-feeding arthropods produce a peritrophic matrix (PM). As mentioned previously, the PM is an acellular sheath that surrounds the blood meal following arthropod feeding and is composed of chitin fibrils and other proteins, including chitin-binding proteins called peritrophins [34, 35]. The PM is analogous, in some respects, to the intestinal mucosa of mammals in that it prevents direct contact of microbes with the epithelium and protects epithelial cells from abrasive food particles. However, the PM is thought to have other important roles such as heme sequestration in *Aedes aegypti* or alteration of the temporal kinetics of the tsetse fly epithelial immune response [93, 94]. Peritrophins are frequently expressed in response to a blood meal; however, some are upregulated in response to commensal microbes or ingestion of pathogens [95, 96]. Adult fleas are not believed to produce a PM based on lack of visual evidence following dissection and imaging of *C*. *felis* or *X*. *cheopis* flea guts at various times after feeding [60, 97].

**Fig 4:**
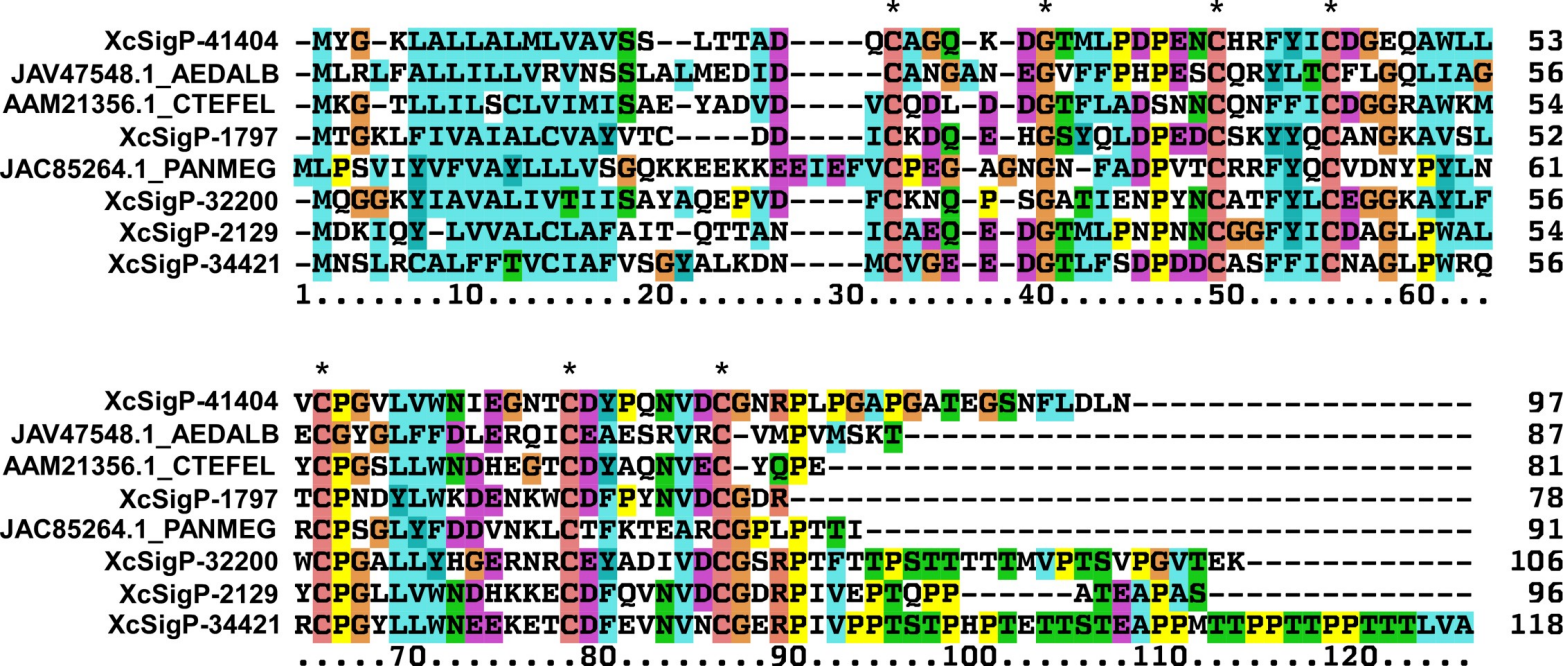
Alignment of differentially regulated *X*. *cheopis* peritrophin-like genes with those of other insects. Multiple sequence alignment of *X*. *cheopis* peritrophins (XcSigP-41404, -1797, -32200, -2129, and -34421; Table 5) with peritrophin sequences of *A*. *albopictus* (JAV47548.1), *C*. *felis* (AAM21356.1), and *P*. *megistus* (JAC85264.1). Asterisks highlight conserved cysteine residues of the peritrophin A domain.

Three peritrophin transcripts were uniquely more highly expressed in response to infection, and another 3 were similarly expressed in response to a sterile or infectious blood meal (Fig 3 and Table 3). Only peritrophin sequences that contained a start codon and were roughly the size of other known peritrophins were selected for multiple sequence alignment (Fig 4 and Table 5). The 5 selected *X*. *cheopis* peritrophin sequences, which share similarity with *C*. *felis* peritrophins, vary from 75 to 118 amino acids and, contain a single PFAM CBM_14, chitin binding Peritrophin-A domain. We compared these sequences to similar proteins in the GenBank database having a maximum length of 150 amino acids and having the CBM_14 motif. The bootstrapped alignment was not very informative, as it contained many clades with weak bootstrap support. Interestingly, many of these single CBM_14 proteins were described from transcriptomes of non-midgut tissues, including a cat flea protein found mostly expressed in Malpighian tubules, hindgut, and trachea [97]. The alignment of five *X*. *cheopis* proteins with analogous proteins of the cat flea, the kissing bug *Panstrongylus megistus* [98], and the mosquito *Aedes albopictus* [99] shows the conserved six cysteines of the CBM_14 motif (Fig 4). Many of these proteins have carboxy-terminal tails rich in Ser and Thr residues that are targets of mucin- type glycosylation as predicted by the NetOglyc server [100].

**Table 5:** Peritrophin transcripts significantly upregulated in response to feeding and infection.

Contig Name	Encoded Protein	Fold Change	E Value	Coverage (%)	Protein Database
XcSigP-32200*	Peritrophin-like protein 3	345	3e-010	98	INSECTA
XcSigP-1797*	Peritrophin-1-like Protein	10.8	8e-010	69	INSECTA
Xc26330*	Peritrophic membrane protein 4	7.8	6e-025	46	INSECTA
XcSigP-34421	Peritrophin-like protein 3	7.2	6e-011	89	NR-LIGHT
XcSigP-2129	Peritrophin-like protein 3	4.2	3e-15	90	INSECTA
XcSigP-41404	Peritrophin-like protein 3	2.9	6e-15	95	INSECTA

*More highly expressed in infected fleas than in sterile blood-fed fleas (Fig 3)

Since *X*. *cheopis* purportedly does not make a peritrophic matrix, and this family of proteins is produced by non-midgut tissues, it is possible that these proteins are associated with chitin-containing structures, such as trachea, as shown for *C*. *felis*, or salivary ducts. The peritrophins could also be associated with the proventricular spines. The putative mucin rich domain of these proteins may function as a mucin coat on top of otherwise exposed chitin structures.

### Analysis of putative peritrophins and peritrophic matrix

Because multiple peritrophins, a trehalose transporter potentially indicative of early chitin biosynthesis, and extracellular matrix genes were upregulated in response to blood-feeding, we wanted to verify that *X*. *cheopis* does not produce a PM (Figs 2C and 3, Table 3). It is often unequivocally stated that fleas do not produce a PM, but few studies are available to support this assertion. Some rely on its visual absence by microscopy 2, 6, or 18h following blood feeding when compared to sand flies and mosquitoes, but do not show images of the negative result [60]. Blood-feeding arthropods that feed multiple times per day, such as cat fleas and body lice, which rapidly digest and turnover their midgut contents following feeding, do not produce a PM and their peritrophins may be purposed for other functions [60, 97, 101, 102]. The rapid rate of digestive processing for these frequently feeding insects may exert selection pressure against utilization of a PM; but *X*. *cheopis*, which feeds less often, might differ in this respect. In addition, PM formation and degradation is known to be temporal, the timing of which can be variable even between closely related insect species [103]. For these reasons, we felt it was important to re-examine whether *X*. *cheopis* produce a PM in response to a blood meal.

**Fig 5:**
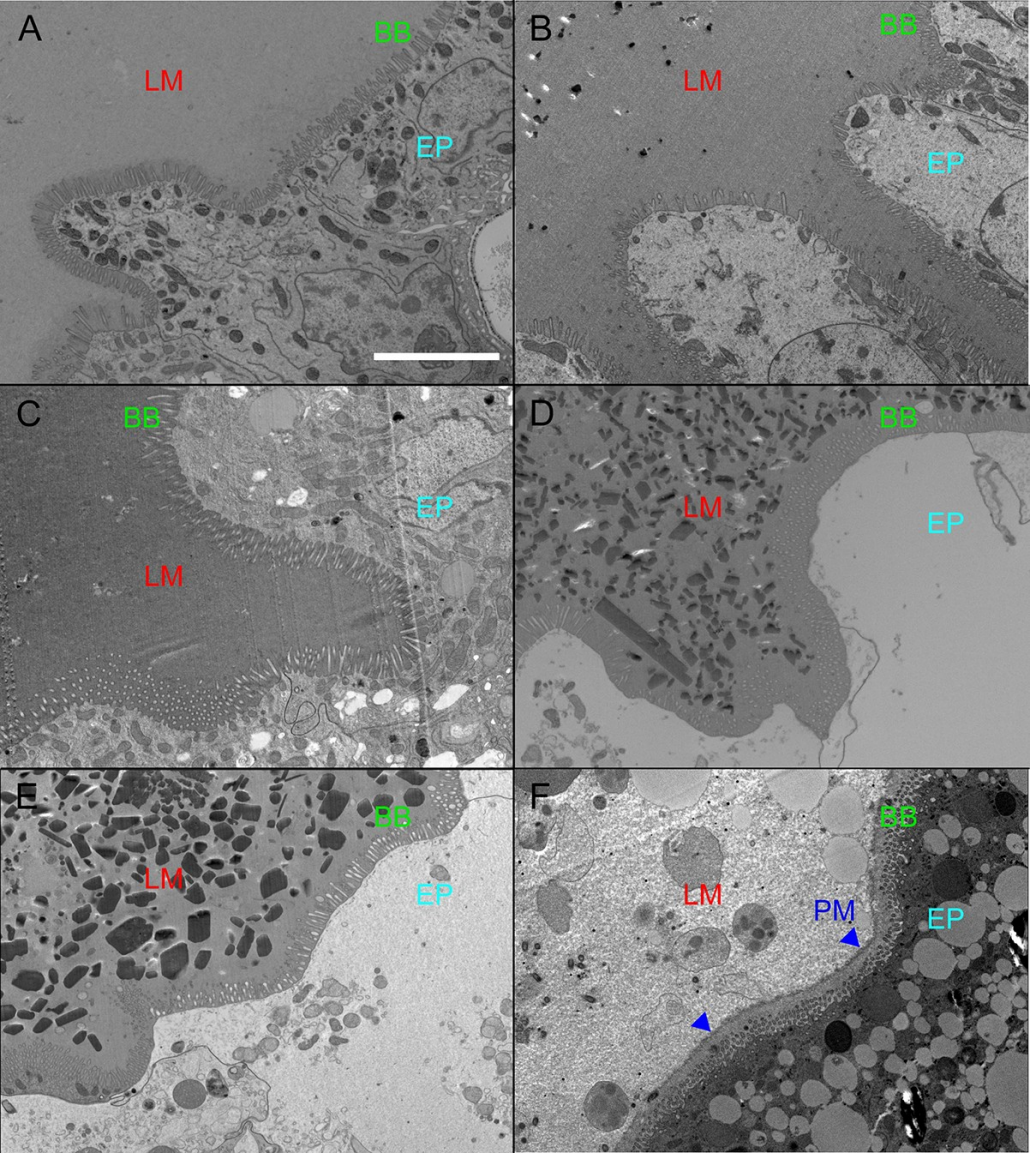
Absence of a detectable peritrophic matrix in the *X*. *cheopis* midgut following blood feeding. Electron micrographs of the *X*. *cheopis* midgut epithelium either A) 0, B) 4, C) 8, D) 12, or E) 24h after blood feeding; or F) the gut of an *I*. *scapularis* nymph 72 h following host attachment. An obvious peritrophic matrix is seen in *I*. *scapularis* but not *X*. *cheopis*. Lumen (LM), Brush border (BB), Epithelium (EP), Peritrophic matrix (PM). Images are representative of the digestive tracts of 3 female fleas (4 fields/flea) dissected and fixed following a sterile blood meal. Scale bar = 5 μm.

To address this, groups of *X*. *cheopis* were fed sterile blood and their guts were excised at 0, 2, 4, 8, 12, and 24h post-feeding and then fixed, sectioned, and stained with either H&E or DAPI and wheat germ agglutinin (WGA) conjugated to Alexa Fluor-488 (S3 Fig). WGA binds to N-acetylglucosamine and N-acetylneuraminic acid residues present in chitin, an important component of the arthropod PM. The flea’s proventricular spines are lined with chitin, thus serving as an internal positive control for WGA staining in our sectioned digestive tract samples (S3A Fig). Faint fluorescence at the border of the gut epithelium and lumen was observed, indicating that if *X*. *cheopis* does produce a PM it is very thin (S3B Fig). Transmission electron micrographs of *X*. *cheopis* midguts showed the same results as the WGA staining experiments, possibly displaying pieces of a very thin structure (Fig 5D). Fleas showed no evidence of thick PM formation at either 0, 4, 8, 12, or 24h following a blood meal (Fig 5A–5E). In contrast, *Ixodes scapularis* nymphs (our positive control) clearly showed a thick PM layer separating the lumen from the midgut epithelium 72h after feeding (Fig 5F). If *X*. *cheopis* does make a PM during the first 24h following a blood meal, it is very thin, delicate, and ephemeral compared to the PM of ticks, mosquitoes, and sandflies.

### Distribution of peritrophin transcripts in flea tissues

Certain internal structures of insects, such as trachea and Malpighian tubules, are surrounded by a chitinous cuticle. The flea proventriculus is also chitinous and WGA staining of the proventriculus was observed in sectioned flea guts (S3A Fig). Although flea Malpighian tubules are relatively large and easy to excise from the rest of the digestive tract, the tracheal system consists of a series of branching tubes, which gradually give rise to finer tubes, the tracheoles, that are distributed throughout the insect and are essential for gas exchange [104]. During flea dissection, the tracheal network is damaged, occasionally leaving small, thin portions of tracheal tissue superficially attached to the midgut (S4A and S4B Fig). Though this tissue was selectively reduced during excision and isolation of flea digestive tracts, we suspect a small amount of tracheal tissue was present in the samples used for RNA extraction. Based on the presence of chitin in flea trachea and the similarity of our peritrophin sequences to those isolated from non-midgut tissues, we hypothesized these peritrophin transcripts may derive primarily from the flea trachea.

To determine tissue specific expression of flea peritrophins, two peritrophin transcripts, peritrophin A (XcSigP-41404) and peritrophin B (XcSigP-32200; Table 5), were selected for *in situ* hybridization and RT-qPCR analysis based on their abundance and change in expression in response to blood-feeding and infection. Peritrophin A was upregulated 3-fold in response to feeding and was the 21^st^-most abundant gene transcript (FPKM = 475) upregulated in response to blood-feeding (Table 5 and S3 Table). Peritrophin B was less abundant (FPKM = 7); however, it was upregulated in response to blood-feeding and moreso in infected fleas (Fig 3 and Table 3).

**Fig 6:**
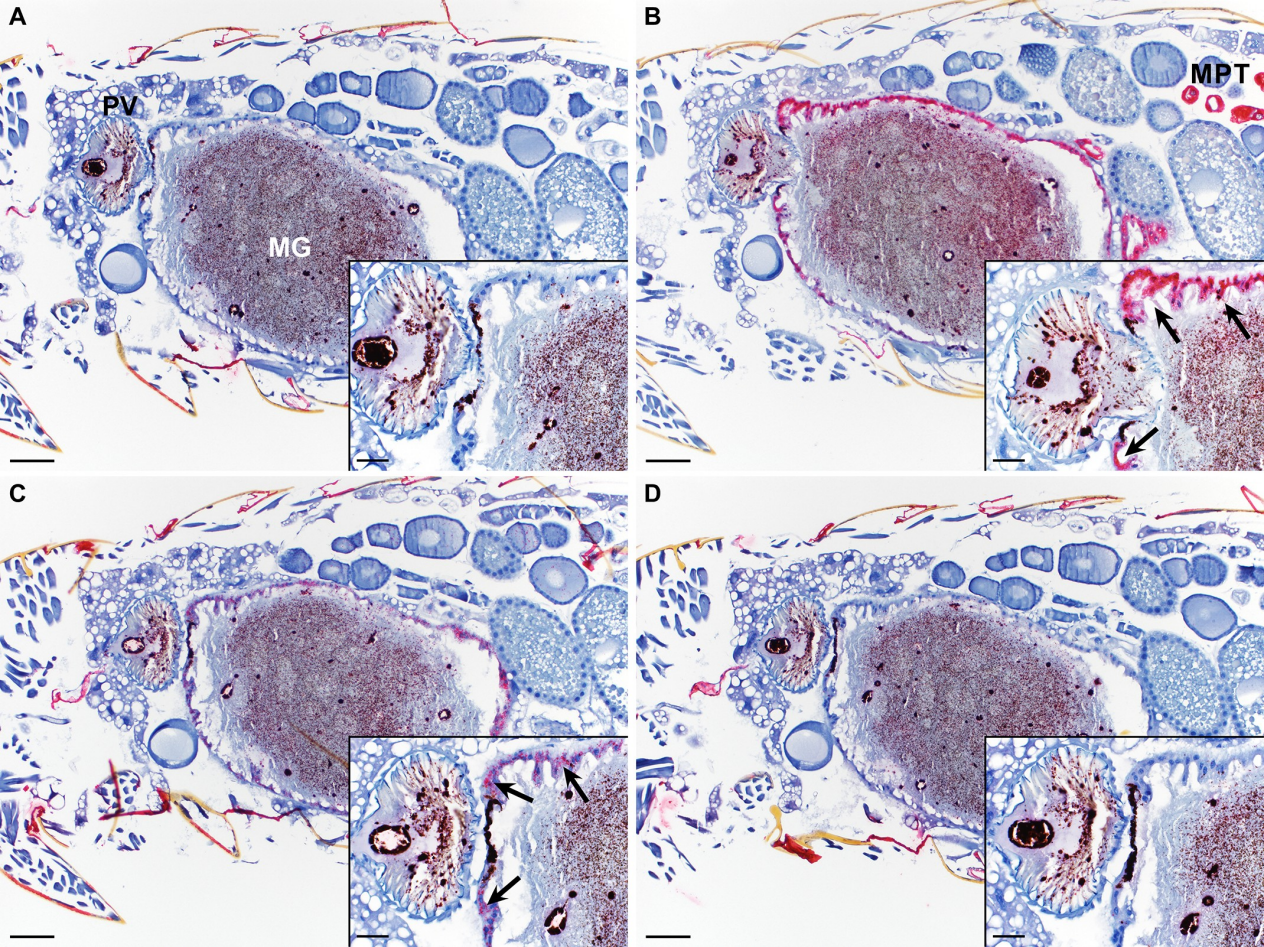
Detection of peritrophin transcripts in tissues of infected fleas using *in situ* hybridization. Fixed sections of whole fleas infected with KIM6+ *Y*. *pestis* were hybridized to probes with specificity for 1 of 4 different mRNA transcripts: **A)** Ajuba (mouse-specific transcript; negative control), **B)** a trypsin-like serine protease (XcSigP-74269; positive control), **C)** peritrophin A (XcSigP-41404), or **D)** peritrophin B (XcSigP-32200). Tissues stain red (Fast Red dye) where transcript was detected. The proventriculus (PV) and midgut (MG) epithelium are magnified in the inset images in the bottom right corner of each panel and arrows highlight areas of strong (trypsin like-serine protease; panel B) or moderate multifocal (Peritrophin A; panel C) staining in the midgut epithelia. (MPT) Malpighian tubules. Images are representative of groups of 6 fleas per transcript. Non-specific staining of the flea cuticle was detected but occurred equivalently across all treatment groups. Slides were counterstained with hematoxylin (blue). Scale bars for larger images = 50 μm, inset images = 20 μm.

Infected whole fleas were collected 4h after feeding, fixed and sectioned, and flea transcripts were detected by hybridization to RNA probes. Flea sections were screened for expression of 4 transcripts: a trypsin-like serine protease (XcSigP-74269, midgut epithelium positive control), peritrophin A, peritrophin B, and Ajuba (a mouse-specific transcript, negative control). Sections stained using the serine protease positive control showed strong staining in the midgut epithelium and the Malpighian tubules, while no transcript was detected in the negative control slides (Fig 6A and 6B). For peritrophin A, moderate multifocal staining was observed only in the flea midgut epithelium and was absent from the chitinous proventriculus (Fig 6C). Peritrophin B transcript was not detectable in any of the stained flea sections, suggesting its expression in the digestive tract is limited, if it is expressed in these tissues at all (Fig 6D). Unfortunately, we were unable to capture enough tracheal tissue during flea sectioning in order to evaluate peritrophin expression in this tissue.

To better assess peritrophin expression in flea tracheal tissue and confirm the results of the hybridization experiment RT-qPCR was used on RNA isolated from 3 groups of tissue: 1) midguts and proventriculus; 2) trachea; and 3) pooled Malpighian tubules and hindguts excised from fleas 4h after ingesting sterile blood. We found that both peritrophins were at least modestly expressed in all 3 tissue samples (S4C and S4D Fig). Peritrophin A transcript was 4-fold more abundant in midgut/proventricular tissue following flea blood-feeding (S4C Fig). Peritrophin B was expressed at slightly higher levels in both midgut/proventriculus and tracheal samples, compared to the hindgut/Malpighian tubule samples, but was detected in all 3 tissue groupings (S4D Fig). Because the elongation factor-1 delta (*ef-1d*) housekeeping gene used for normalization was selected based on its consistent expression pattern in midgut and proventriculus tissue and *ef-1d* expression could vary between tissue types, we tested two other housekeeping genes: enolase (*eno*) and UDP-glucose glycoprotein glucosyltransferase isoform 2 (*uggt2*; S2 Table). Ultimately, the expression pattern was similar, regardless of the housekeeping-gene used, for peritrophin A (S4C, S4E and S4G Fig). However, while not statistically significant, peritrophin B expression was roughly 2-fold higher in 2 of the 3 tracheal tissue samples compared to MG-PV or HG-MPT tissue when using *eno* or *uggt2* for expression normalization (S4F and S4H Fig). The collective results of the tissue specificity experiments indicate that peritrophin A is most highly expressed in the midgut epithelium and peritrophin B is expressed at low levels in all 3 tissue groupings, but suggest higher levels of expression in the trachea.

Due to increased expression of peritrophin A in the midgut following a blood meal and poor evidence of a substantial PM we propose this transcript plays a role unrelated to generation of the PM. This peritrophin may not bind chitin or could be a non-functional evolutionary leftover from when fleas diverged from the Mecoptera (scorpion flies) [105], some species of which produce a PM [106]. In contrast, peritrophin B may be more highly expressed in tracheal tissue following *Y*. *pestis* infection. Fleas open and close their spiracles more frequently during periods of high metabolism, such as during blood digestion, inflating and deflating the tracheal system to distribute oxygen [107]. Upon inflation, the epithelial layer of the trachea is stretched [108]. The rapid pulsations of the trachea could require repair and/or replacement of the tracheal cuticle; potentially explaining expression of some flea peritrophins following ingestion of a blood meal (Table 5). *Y*. *pestis* may induce increased metabolism and tracheal pulsation rates in infected fleas that could require expression of certain peritrophins to repair the tracheal cuticle.

### Summary

Overall, differential regulation of such a small subset of the flea transcriptome (23 annotated and 11 uncharacterized genes) in response to oral infection suggests that *Y*. *pestis*, similarly to its pathogenesis strategy in the mammalian host [109], has evolved mechanisms to reduce detection and avoid elimination by the insect host. After being taken up in a blood meal, the majority of the individual planktonic *Y*. *pestis* begin to coalesce in the flea digestive tract and rapidly form dense multicellular aggregates, masses, or “casts” [3, 11]. The aggregates are coated with an amorphous brown-colored viscous material that probably derive from blood meal components, including proteins and lipids, and potentially flea-derived components as well [12, 110]. This material masks the bacteria from direct interaction with the digestive tract epithelium and may obscure the bacterial signals that induce the flea response to infection. In addition, the bacteria enclosed within an aggregate may be shielded from the flea antibacterial components that are generated. Rapid aggregation has other adaptive effects as well: it represents the initial stage of biofilm development in the flea, which is required for efficient transmission; and the formation of large aggregates may inhibit clearance of infection by defecation.

The early immune response of the flea gut to infection with *Y*. *pestis* primarily involves production of antimicrobial peptides and a serine protease likely regulated by the IMD pathway. The microbial-induced ROS response of fleas to bacterial infection appears to be limited. Differential expression of transcripts related to chemosensation and metabolism in fleas challenged with *Y*. *pestis* are suggestive of early behavioral and physiological changes, such as feeding, host-seeking, and blood meal processing for infected fleas and a role for modulation of lipid and carbohydrate metabolism in chronic flea infection. These previously unknown alterations to expression of behavioral and metabolic genes may affect retention, survival, and transmission of plague bacilli from the gut. Future sequencing of medically relevant flea genomes will allow researchers to more directly address the role of pathogen-responsive flea transcripts using RNAi gene silencing and, potentially, genetic modification of fleas.

## Supporting information

S1 Fig Bioanalyzer analysis of flea RNA. Purified RNA from all 18 *X*. *cheopis* digestive tract samples (6 each from unfed, sterile blood-fed, and infected fleas) was evaluated using an Agilent 2100 Bioanalyzer. Electropherogram patterns are indicative of high-quality RNA with no evidence of degradation. L = RNA Ladder.
(TIF)

S2 Fig Validation of RNA-seq gene expression data by RT-qPCR. A) Relative expression of 6 flea transcripts in digestive tract RNA samples in response (4h post-feed) to sterile blood-feeding, infection with *Y*. *pestis*, or starvation (unfed). Each symbol shows gene expression relative to the flea elongation factor 1-delta (ef1δ) transcript in 5–6 independent PCR reactions for each of the sterile blood-fed, infected, and unfed flea RNA samples. Bars indicate the mean and standard deviation. B) Linear regression of log2-fold expression values for the 6 flea transcripts in unfed and infected digestive tract samples (n = 11–12) normalized to average expression values for sterile blood fed samples for both RNA-seq and RT-qPCR analysis (R^2^ = 0.849).
(TIF)

S3 Fig *X*. *cheopis* does not produce a thick peritrophic matrix during the first 24 hours following feeding. A) Digestive tract sections for an unfed flea or B) from fleas dissected 2, 4, 8, 12, or 24h post-feeding (HPF), left panels show fluorescent microscopy images of sections stained with stained with wheat germ agglutinin conjugated to Alexafluor-488 (WGA-AF) and DAPI. Right panels show light microscopy images of sections stained with H&E. The chitinous proventriculus stains strongly with WGA (green color) and served as an internal positive control for staining of chitin. Images are representative of sections of 6 to 8 female flea guts per timepoint, pulled from a group of *X*. *cheopis* fed sterile rat blood.
(TIF)

S4 Fig Detection of peritrophins in flea tissues using RT-qPCR. A, B) Light microscopy images of tissues associated with the *X*. *cheopis* digestive tract: hindgut (HG), midgut (MG), Malpighian tubules (MPT), proventriculus (PV), and trachea (TR). Boxed region in image A is magnified and shown in image B. Relative expression of peritrophin A (C, E, G; XcSigP-41404) and peritrophin B (D, F, H; XcSigP-32200) transcripts in RNA samples extracted from 3 different sets of flea tissue: 1) hindgut and malpighian tubules (HG-MPT); 2) midgut and proventriculus (MG-PV); and 3) trachea (TR). Each symbol shows gene expression relative to (C, D) flea elongation factor 1-delta (*ef-1d*), (E, F) enolase (*eno*), or (G, H) UDP-glucose glycoprotein glucosyltransferase isoform 2 (*uggt2*) transcripts in 3 independent PCR reactions for each of the tissue groupings. RNA samples were extracted from tissues isolated and pooled from groups of 10 female *X*. *cheopis* 4h after ingesting sterile blood in 3 independent experiments. The mean and standard deviation are indicated. * p < 0.01 by one-way ANOVA with Tukey’s post-test.
(TIF)

S1 Table Flea infection summary. (DOCX)

S2 Table qPCR primer and probe sequences. (DOCX)

S3 Table The 25 most abundant (highest FPKM values) significantly altered transcripts with ≥2-fold change in expression in response to feeding and infection. (DOCX)

S4 Table Infection-responsive hypothetical and uncharacterized gene transcripts. (DOCX)

S5 Table Oxidant metabolism/detoxification transcripts significantly altered with ≥2-fold change in expression in response to feeding and infection. (DOCX)
