# Supplementary figures and images for: Transcriptomic profiling of the digestive tract of the rat flea, Xenopsylla cheopis, following blood feeding and infection with Yersinia pestis
Source: PLoS Negl Trop Dis. 2020 Sep 18;14(9):e0008688. doi: 10.1371/journal.pntd.0008688 (PMC7526888; doi:10.1371/journal.pntd.0008688)

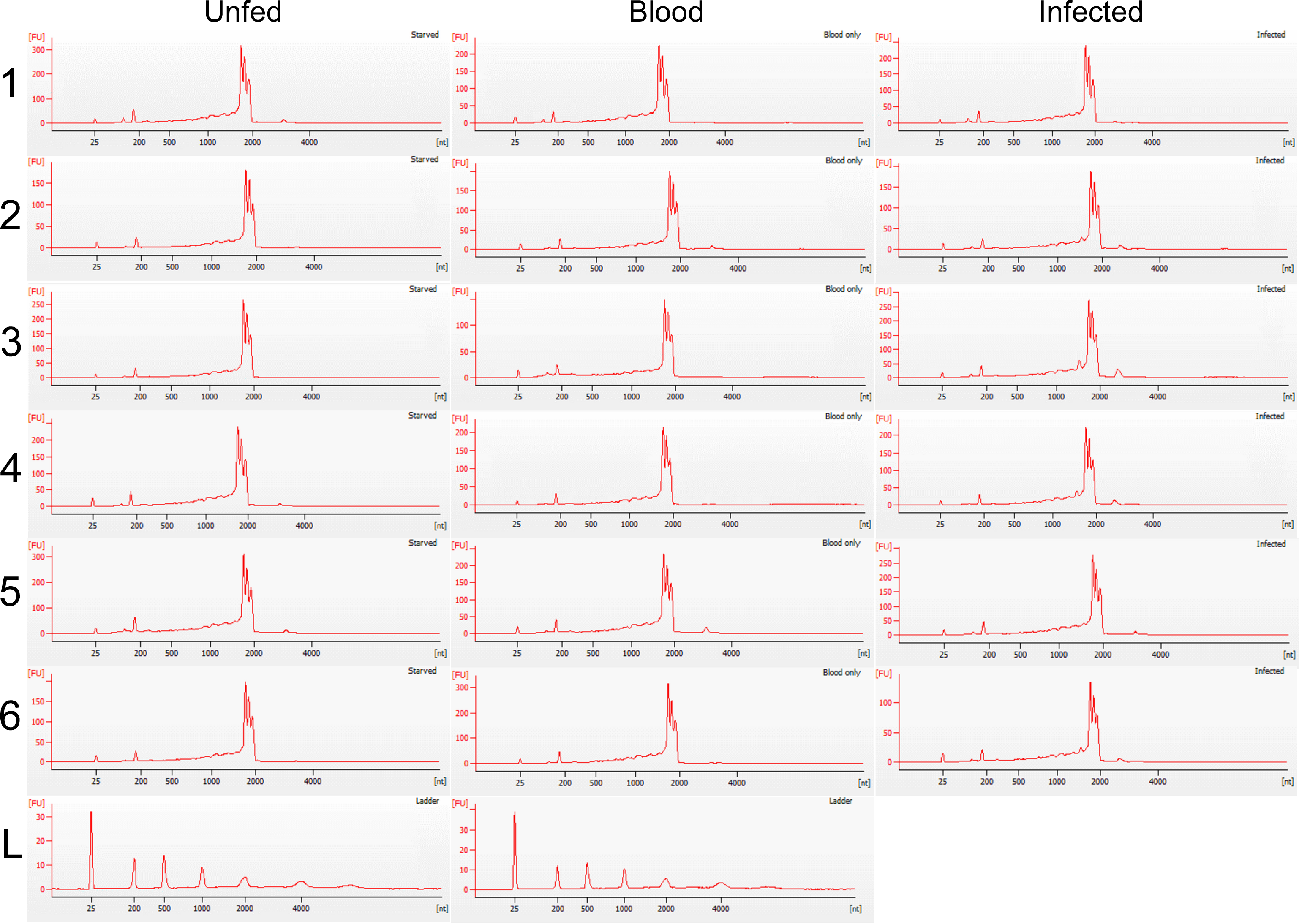

Supplement: S1 Fig — Purified RNA from all 18 X. cheopis digestive tract samples (6 each from unfed, sterile blood-fed, and infected fleas) was evaluated using an Agilent 2100 Bioanalyzer. Electropherogram patterns are indicative of high-quality RNA with no evidence of degradation. L = RNA Ladder. (TIF) [file pntd.0008688.s001.tif]

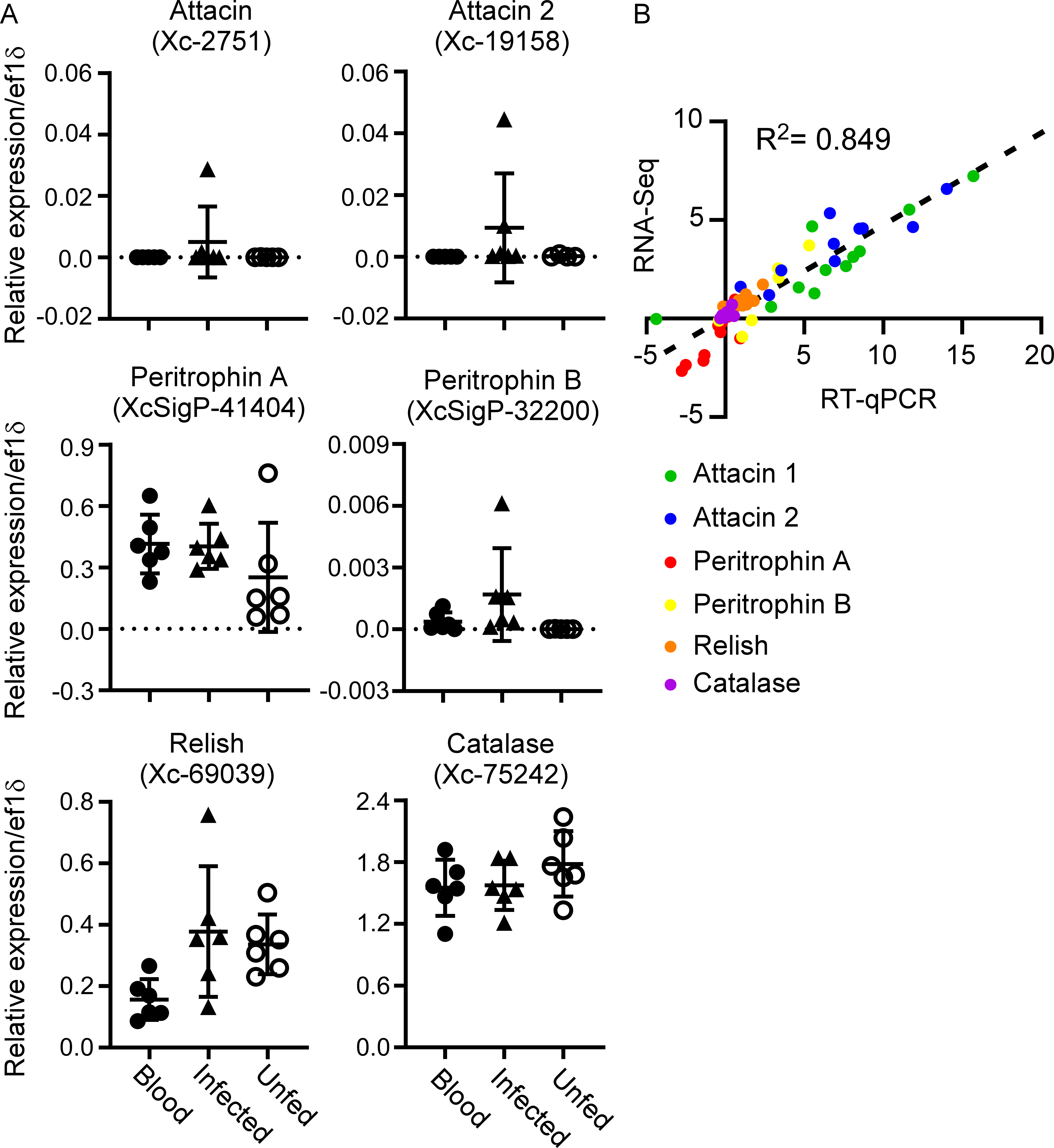

Supplement: S2 Fig — A) Relative expression of 6 flea transcripts in digestive tract RNA samples in response (4h post-feed) to sterile blood-feeding, infection with Y. pestis, or starvation (unfed). Each symbol shows gene expression relative to the flea elongation factor 1-delta (ef1δ) transcript in 5–6 independent PCR reactions for each of the sterile blood-fed, infected, and unfed flea RNA samples. Bars indicate the mean and standard deviation. B) Linear regression of log2-fold expression values for the 6 flea transcripts in unfed and infected digestive tract samples (n = 11–12) normalized to average expression values for sterile blood fed samples for both RNA-seq and RT-qPCR analysis (R2 = 0.849). (TIF) [file pntd.0008688.s002.tif]

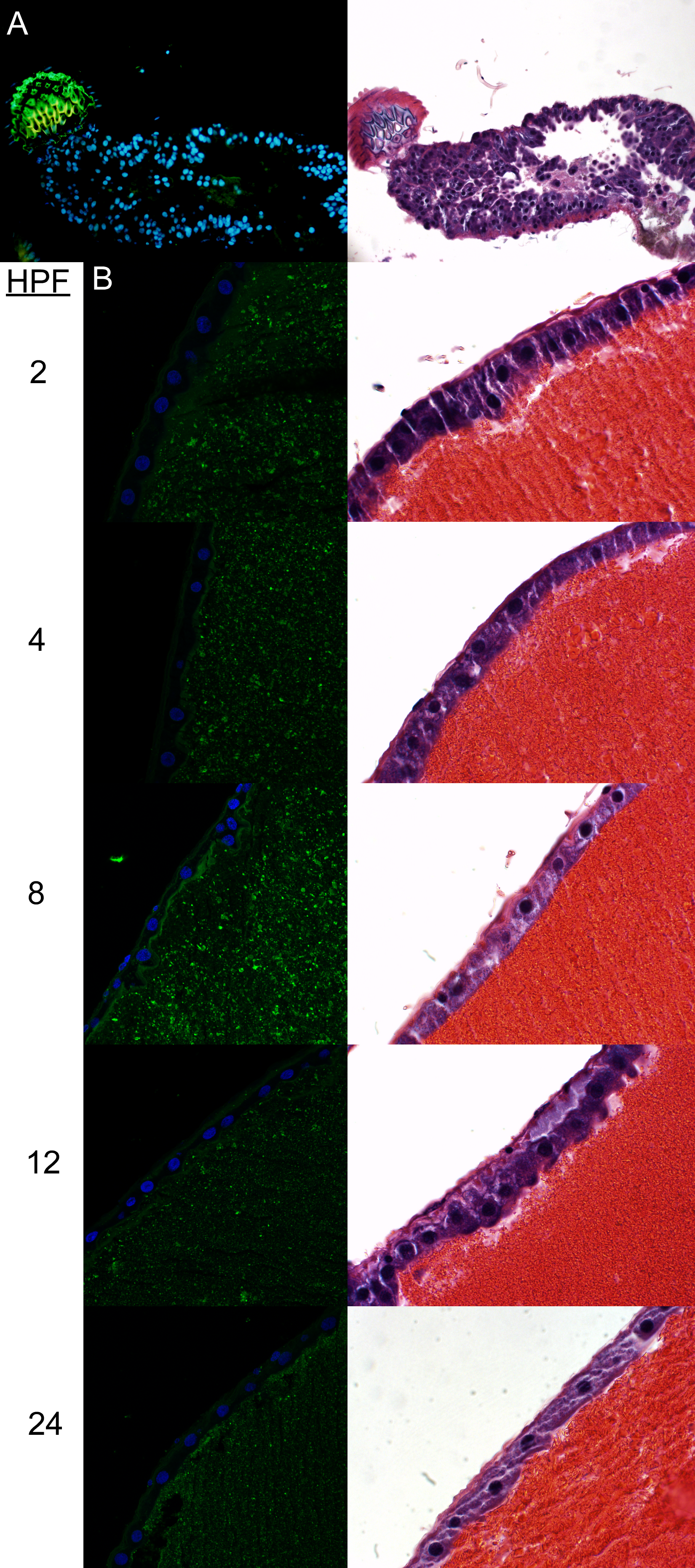

Supplement: S3 Fig — A) Digestive tract sections for an unfed flea or B) from fleas dissected 2, 4, 8, 12, or 24h post-feeding (HPF), left panels show fluorescent microscopy images of sections stained with stained with wheat germ agglutinin conjugated to Alexafluor-488 (WGA-AF) and DAPI. Right panels show light microscopy images of sections stained with H&E. The chitinous proventriculus stains strongly with WGA (green color) and served as an internal positive control for staining of chitin. Images are representative of sections of 6 to 8 female flea guts per timepoint, pulled from a group of X. cheopis fed sterile rat blood. (TIF) [file pntd.0008688.s003.tif]

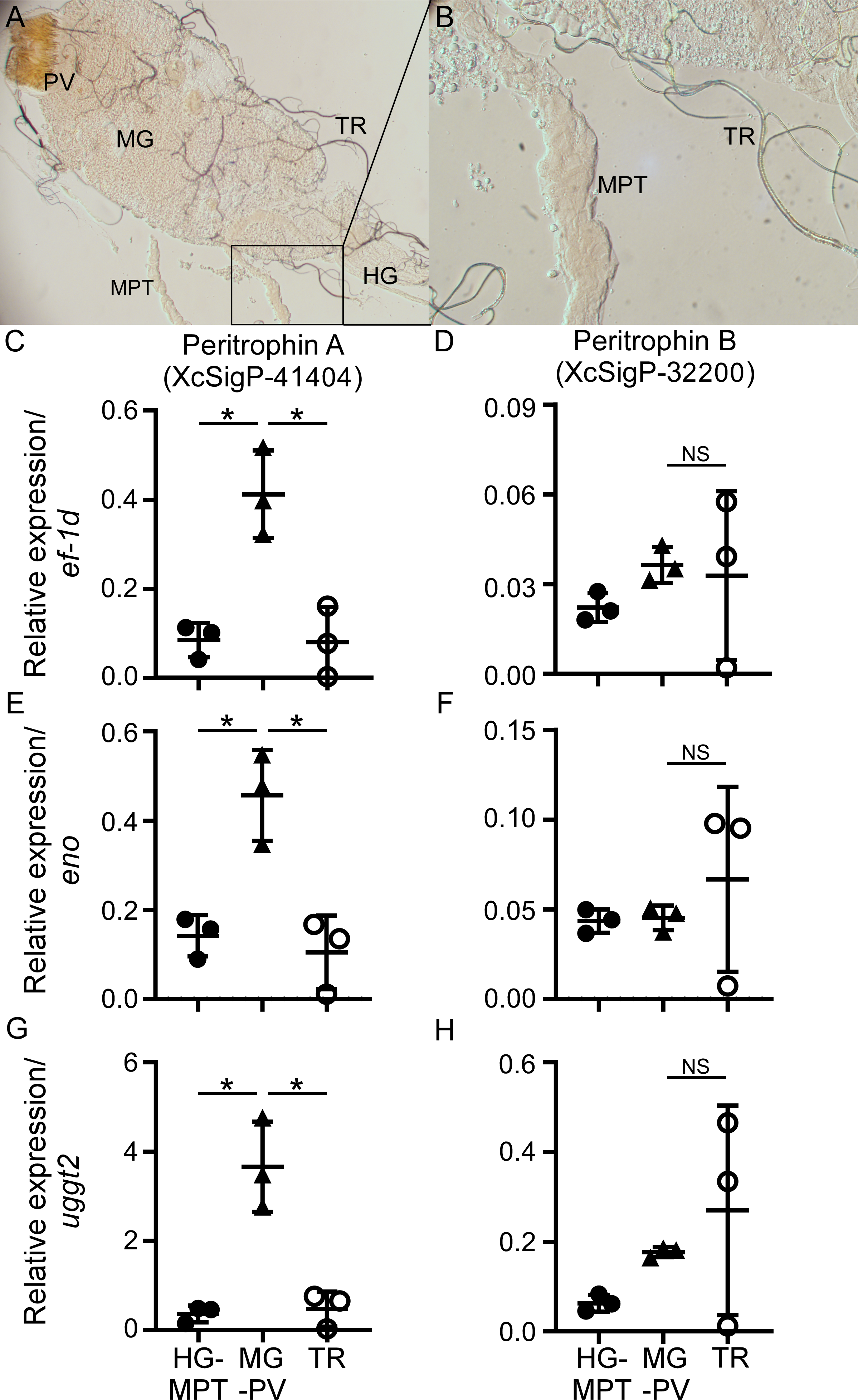

Supplement: S4 Fig — A, B) Light microscopy images of tissues associated with the X. cheopis digestive tract: hindgut (HG), midgut (MG), Malpighian tubules (MPT), proventriculus (PV), and trachea (TR). Boxed region in image A is magnified and shown in image B. Relative expression of peritrophin A (C, E, G; XcSigP-41404) and peritrophin B (D, F, H; XcSigP-32200) transcripts in RNA samples extracted from 3 different sets of flea tissue: 1) hindgut and malpighian tubules (HG-MPT); 2) midgut and proventriculus (MG-PV); and 3) trachea (TR). Each symbol shows gene expression relative to (C, D) flea elongation factor 1-delta (ef-1d), (E, F) enolase (eno), or (G, H) UDP-glucose glycoprotein glucosyltransferase isoform 2 (uggt2) transcripts in 3 independent PCR reactions for each of the tissue groupings. RNA samples were extracted from tissues isolated and pooled from groups of 10 female X. cheopis 4h after ingesting sterile blood in 3 independent experiments. The mean and standard deviation are indicated. * p < 0.01 by one-way ANOVA with Tukey’s post-test. (TIF) [file pntd.0008688.s004.tif]
