# Supplementary material for: Transcriptomic profiling of the digestive tract of the rat flea, Xenopsylla cheopis, following blood feeding and infection with Yersinia pestis
Source: PLoS Negl Trop Dis. 2020 Sep 18;14(9):e0008688. doi: 10.1371/journal.pntd.0008688 (PMC7526888; doi:10.1371/journal.pntd.0008688)
Supplement: S1 Table — (DOCX) [file pntd.0008688.s005.docx]

**S1 Table. Flea infection summary**

| Sample/  Experiment | Concentration of KIM6+ *Y. pestis* in the Infectious Blood Meal (CFU/ml) | Median CFU/Flea  (T=0) | Range | n |
| --- | --- | --- | --- | --- |
| Infected #1 | 6.5 x 10^8^ | 4.3 x 10^4^ | 1.3 x 10^4^ – 9 x 10^4^ | 18 |
| Infected #2 | 2.9 x 10^8^ | 1.1 x 10^4^ | 1.1 x 10^3^ – 2.3 x 10^4^ | 20 |
| Infected #3 | 4.6 x 10^8^ | 1.6 x 10^5^ | 1.1 x 10^5^ –2.8 x 10^5^ | 20 |
| Infected #4 | 3.3 x 10^8^ | 5.0 x 10^4^ | 4.0 x 10^2^ –7.3 x 10^4^ | 20 |
| Infected #5 | 5.7 x 10^8^ | 7.4 x 10^4^ | 1.1 x 10^3^ –1.5 x 10^5^ | 20 |
| Infected #6 | 3.7 x 10^8^ | 4.0 x 10^4^ | 1.6 x 10^3^ – 1.1 x 10^5^ | 20 |
| Combined | 4.5 x 10^8^ | 5.2 x 10^4^ | 4.0 x 10^2^ –2.8 x 10^5^ | 118 |
