# Supplementary material for: Transcriptomic profiling of the digestive tract of the rat flea, Xenopsylla cheopis, following blood feeding and infection with Yersinia pestis
Source: PLoS Negl Trop Dis. 2020 Sep 18;14(9):e0008688. doi: 10.1371/journal.pntd.0008688 (PMC7526888; doi:10.1371/journal.pntd.0008688)
Supplement: S2 Table — (DOCX) [file pntd.0008688.s006.docx]

**S2 Table. qPCR primer and probe sequences**

| **Gene/Product** | **ERGO/GenBank#** | **Type** | **5'-3' sequence** |
| --- | --- | --- | --- |
| Peritrophin A | XcSigP-41404 | For | GGTCAAAAAGATGGAACAATGCTT |
|  |  | Rev | GGACATACTAAGAGCCACGCCT |
|  |  | FAM-BHQ1 | CTGACCCAGAGAACTGCCACCGATTC |
| Peritrophin B | XcSigP-32200 | For | CTTCTGGTGTCCAGGTGCACTT |
|  |  | Rev | GTTGGCCTGCTACCACAATCT |
|  |  | FAM-BHQ1 | TGCATATTCACATCTGTTCCTTTCTCCGTG |
| Attacin | Xc2751 | For | GTTGGTGGCGGTTTACAATATTCT |
|  |  | Rev | CATCCAGACTTGAACGACGATCT |
|  |  | FAM-BHQ1 | TGGCACCACCAGTTGCACCCA |
| Attacin 2 | Xc19158 | For | ATGCTAATTGGGCGCGTACTT |
|  |  | Rev | CCAACATCAGTGCCAAAACCT |
|  |  | FAM-BHQ1 | CGGCTTGCAATACACTCACGACAGGG |
| Relish | Xc69039 | For | TCTGGTCCAGCCACAATTTCTT |
|  |  | Rev | TTGCAGTGTGTATAATCCCCATTC |
|  |  | FAM-BHQ1 | CCCACTCGCATCATCTCGTCCTCAA |
| Catalase | Xc75242 | For | AGACTGATCAAGGCATCAAAAACCT |
|  |  | Rev | ACGGATTGAATAATCAGGATCAGTAC |
|  |  | FAM-BHQ1 | CCGATCGCGCCGACCAGC |
| Enolase  (*eno*) | Xc39078 | For | GATTTGGTGACTGACTTAGGCCTT |
|  |  | Rev | CGAGACTCCTTTACCCATATATTGACTC |
|  |  | CFG-BHQ1 | AGCTGCCGTTCCTTCTGGTGCTTCC |
| Alpha-aminoadipic  semialdehyde dehydrogenase  (*aldh7a1*) | Xc19393 | For | CGTTTGCTGCGGTGCTT |
|  |  | Rev | TTGTTGACCAACGTGAGTGCTT |
|  |  | CFG-BHQ1 | CAGCTATGGCTAAAGACACGCGCGTC |
| Elongation factor 1 delta  (*ef-1d*) | Xc74700 | For | ATTCACCAATCTTCCCAGAACTTC |
|  |  | Rev | GCTCGGCAACATATTAAAAATTCCT |
|  |  | CFG-BHQ1 | TCGAAGCCATAGCTGCAATACCATCCA |
| UDP-glucose:glycoprotein  glucosyltransferase isoform X2  (*uggt2*) | Xc50242 | For | AGTTCTAGGCGAAAAGGCCTT |
|  |  | Rev | CAGGTTGCGGTGAGTCATCTT |
|  |  | CFG-BHQ1 | TTTGTAGCTCGACACCATATCCAGAGAGCC |
