# Supplementary material for: Transcriptomic profiling of the digestive tract of the rat flea, Xenopsylla cheopis, following blood feeding and infection with Yersinia pestis
Source: PLoS Negl Trop Dis. 2020 Sep 18;14(9):e0008688. doi: 10.1371/journal.pntd.0008688 (PMC7526888; doi:10.1371/journal.pntd.0008688)
Supplement: S3 Table — (DOCX) [file pntd.0008688.s007.docx]

| **Contig Name** | **Encoded Protein** | **Fold Change** | **FPKM** | **E Value** | **Coverage (%)** | **Function** | **Protein Database** |
| --- | --- | --- | --- | --- | --- | --- | --- |
| XcSigP-51820 | Hypothetical Secreted Protein | -2.5 | 4507 | 1000 | 2 | Unknown  /Secreted | INSECTA |
| Xc63307 | Small Heat Shock Protein 22.1 | 10.0 | 4249 | 4e-64 | 88 | Protein Modification | INSECTA |
| Xc69495 | Heat shock protein-like | 89.0 | 2872 | 2e-40 | 103 | Protein Modification | INSECTA |
| Xc23133 | Pyridoxal/pyridoxine/  pyridoxamine kinase | -2.0 | 2729 | 0 | 96 | Intermediate Metabolism | KOG |
| XcSigP-74309 | Acid Sphingomyelinase | 2.8 | 1518 | 0 | 97 | Lipid Metabolism | KOG |
| Xc42367 | Membrane Protein | 2.1 | 1369 | 3e-26 | 57 | Unknown | NR-LIGHT |
| Xc76827 | Phosphatidylinositol Transfer Protein SEC14 | 7.1 | 1009 | 7e-47 | 91 | Lipid Metabolism | KOG |
| XcSigP-27309 | Pyridoxamine 5'-Phosphate Oxidase | 2.7 | 997 | 3e-62 | 87 | Intermediate Metabolism | KOG |
| Xc25177 | Phosphoenolpyruvate Carboxykinase | 30.2 | 915 | 0 | 98 | Energy Metabolism | KOG |
| Xc73203 | Lipid Storage Droplet-2 | 5.4 | 824 | 2e-81 | 89 | Lipid Metabolism | INSECTA |
| Xc43679 | Alpha Crystallins | 83.5 | 779 | 1e-50 | 92 | Protein Modification | KOG |
| XcSigP-13086 | Transcription Activator MBF2 | 5.9 | 771 | 2e-12 | 74 | Transcription Machinery | CDD |
| XcSigP-57288 | Trypsin-like Serine Protease | 4.0 | 750 | 8e-65 | 103 | Digestive Enzyme | SMART |
| XcSigP-2845 | Heat shock 70 kDa Protein Cognate 3 | 9.6 | 674 | 0 | 100 | Protein Modification | INSECTA |
| XcSigP-5226 | Phosphoserine Phosphatase | 3.3 | 669 | 4e-77 | 94 | Amino Acid Metabolism | KOG |
| Xc53113 | Heat Shock Protein 90 | 2.3 | 655 | 0 | 100 | Protein Modification | INSECTA |
| Xc43623 | Enoyl-CoA Hydratase | 2.2 | 564 | 1e-41 | 101 | Lipid Metabolism | INSECTA |
| Xc12143 | Alpha Crystallins | 366.3 | 531 | 2e-41 | 94 | Protein Modification | KOG |
| Xc23655 | Glutamine Synthetase | 8.2 | 530 | 0 | 96 | Amino Acid Metabolism | KOG |
| XcSigP-45412 | Conserved Secreted Protein | -2.4 | 517 | 7e-62 | ND | Unknown  /Secreted | INSECTA |
| XcSigP-41404 | Peritrophin-like Protein 3 | 2.9 | 475 | 6e-15 | 95 | Extracellular Matrix | INSECTA |
| XcSigP-19634 | Vegetative Cell Wall Protein GP1 | 2.0 | 451 | 8e-10 | 107 | Extracellular Matrix | REFSEQ-INVERTEBRATE |
| Xc71311 | Fatty Acid Synthase-like | -2.3 | 424 | 0 | 101 | Lipid Metabolism | REFSEQ-INVERTEBRATE |
| Xc68493 | 3’ Repair Exonuclease | 2.3 | 408 | 2e-43 | 104 | Nucleotide Metabolism | CDD |
| Xc25086 | 10 kDa Heat Shock Protein | 2.0 | 408 | 4e-43 | 100 | Protein Modification | REFSEQ-INVERTEBRATE |

**S3 Table. The 25 most abundant (highest FPKM values) significantly altered transcripts with ≥2-fold change in expression in response to feeding and infection**
