# Supplementary material for: Transcriptomic profiling of the digestive tract of the rat flea, Xenopsylla cheopis, following blood feeding and infection with Yersinia pestis
Source: PLoS Negl Trop Dis. 2020 Sep 18;14(9):e0008688. doi: 10.1371/journal.pntd.0008688 (PMC7526888; doi:10.1371/journal.pntd.0008688)
Supplement: S4 Table — (DOCX) [file pntd.0008688.s008.docx]

**S4 Table. Infection-responsive hypothetical and uncharacterized gene transcripts**

| **Contig Name** | **Encoded Protein** | **Identification method** | **Result** | **E Value** | **Coverage (%)** | **Protein Database** |
| --- | --- | --- | --- | --- | --- | --- |
| Xc68651 | Uncharacterized Protein LOC100679997 | Statistical comparison | Up in infected  fleas | 3e-15 | 75 | INSECTA |
| XcSigP-47090 | Hypothetical Secreted Protein | Outlier Differential | Up in infected  fleas | 1000 | 8 | INSECTA |
| Xc68475 | T-cell Receptor Beta Chain-like | Outlier Differential | Down in infected  fleas | 3e-27 | 98 | REFSEQ-INVERTEBRATE |
| Xc75032 | Unknown Product | Outlier Differential | Down in infected  fleas | - | - | - |
| Xc76691 | Unknown Product | Outlier Differential | Down in infected  fleas | - | - | - |
| XcSigP-1797 | Hypothetical Secreted Protein | Outlier Differential | Down in infected  fleas | 1000 | 11 | INSECTA |
| XcSigP-68868 | Hypothetical Secreted Protein | Outlier Differential | Down in infected | 1000 | 18 | INSECTA |
| Xc4082 | Unknown Product | Outlier Differential | Down in infected  fleas | - | - | - |
| XcSigP-69966 | Hypothetical Protein eai 14870 | Outlier Differential | Up in blood-fed fleas | 0.001 | 92 | INSECTA |
| XcSigP-5088 | Unknown Product | Outlier Differential | Up in blood-fed fleas | - | - | - |
| XcSigP-46773 | Hypothetical Secreted Protein | Outlier Differential | Down in blood-fed fleas | 1000 | 10 | INSECTA |

- **:** value not determined; unknown gene product
