# Supplementary material for: Transcriptomic profiling of the digestive tract of the rat flea, Xenopsylla cheopis, following blood feeding and infection with Yersinia pestis
Source: PLoS Negl Trop Dis. 2020 Sep 18;14(9):e0008688. doi: 10.1371/journal.pntd.0008688 (PMC7526888; doi:10.1371/journal.pntd.0008688)
Supplement: S5 Table — (DOCX) [file pntd.0008688.s009.docx]

**S5 Table. Oxidant metabolism/detoxification transcripts significantly altered with ≥2-fold change in expression in response to feeding and infection**

| **Contig Name** | **Encoded Protein** | **Fold Change** | **E Value** | **Coverage (%)** | **Protein Database** |
| --- | --- | --- | --- | --- | --- |
| XcSigP-71892 | Cytochrome P450 6k1 | 5.8 | 0 | 98 | REFSEQ-INVERTEBRATE |
| Xc32678 | Cytochrome P450 CYP12A2-like | 4.3 | 0 | 102 | REFSEQ-INVERTEBRATE |
| XcSigP-76747 | Cytochrome P450 | 3.1 | 0 | 100 | REFSEQ-INVERTEBRATE |
| Xc68363 | Thioredoxin and Glutathione Reductase | 2.7 | 0 | 101 | CDD |
| XcSigP-68909 | Cytochrome P450 CYP3/CYP5/CYP6/CYP9 subfamily | 2.5 | 2e-25 | 28 | KOG |
| Xc72247 | Cytochrome P450 | -2.0 | 0 | 97 | REFSEQ-INVERTEBRATE |
| XcSigP-44451 | Cytochrome P450 9e2-like | -2.0 | 0 | 98 | REFSEQ-INVERTEBRATE |
| XcSigP-68683 | Cytochrome P450 305a1 | -2.0 | 0 | 99 | REFSEQ-INVERTEBRATE |
| Xc76671 | Cytochrome P450 302a1 | -2.0 | 0 | 99 | REFSEQ-INVERTEBRATE |
| XcSigP-75608 | Cytochrome P450 9f2 | -2.1 | 0 | 96 | REFSEQ-INVERTEBRATE |
| Xc35910 | Cytochrome P450 | -2.5 | 0 | 98 | REFSEQ-INVERTEBRATE |
| XcSigP-2962 | NADPH Cytochrome p450 Reductase | -2.8 | 0 | 101 | CDD |
| Xc27581 | Cytochrome P450 4C1-like | -3.0 | 0 | 95 | REFSEQ-INVERTEBRATE |
| Xc18568 | Cytochrome P450 304a1 | -3.2 | 0 | 100 | REFSEQ-INVERTEBRATE |
| Xc70362 | Cytochrome P450 9e2-like | -4.7 | 0 | 79 | REFSEQ-INVERTEBRATE |
| Xc62977 | Cytochrome P450 | -13.6 | 0 | 88 | REFSEQ-INVERTEBRATE |
